# Beta-3 adrenergic receptor overexpression reverses aortic stenosis–induced heart failure and restores balanced mitochondrial dynamics

**DOI:** 10.1007/s00395-022-00966-z

**Published:** 2022-11-29

**Authors:** Andrés Pun-García, Agustín Clemente-Moragón, Rocio Villena-Gutierrez, Monica Gómez, David Sanz-Rosa, Anabel Díaz-Guerra, Belén Prados, Juan Pablo Medina, Fermí Montó, Maria Dolores Ivorra, Cristina Márquez-López, Alessandro Cannavo, Juan A. Bernal, Walter J. Koch, Valentin Fuster, José Luis de la Pompa, Eduardo Oliver, Borja Ibanez

**Affiliations:** 1 Centro Nacional de Investigaciones Cardiovasculares (CNIC), IIS-Fundación Jiménez Díaz University Hospital, Melchor Fernandez Almagro, 3, 28029 Madrid, Spain; 2 CIBERCV, Madrid, Spain; 3 Universidad Europea de Madrid, Madrid, Spain; 4 Intercellular Signalling in Cardiovascular Development and Disease Laboratory, CNIC, Madrid, Spain; 5 Cardiology Department, IIS-Fundación Jiménez Díaz University Hospital, Madrid, Spain; 6 Departamento de Farmacología, Facultad de Farmacia, ERI BIOTECMED, Universitat de València, Burjassot, Spain; 7 Center for Translational Medicine and Department of Pharmacology, Lewis Katz School of Medicine, Temple University, Philadelphia, PA USA; 8 Department of Translational Medical Sciences, Federico II University of Naples, Naples, Italy; 9 Icahn School of Medicine at Mount Sinai, New York, NY USA; 10 Centro de Investigaciones Biológicas Margarita Salas (CIB), CSIC, Madrid, Spain

**Keywords:** Heart failure, Hypertrophy, Mitochondria, Beta adrenergic system, Metabolism, Aortic stenosis

## Abstract

Aortic stenosis (AS) is associated with left ventricular (LV) hypertrophy and heart failure (HF). There is a lack of therapies able to prevent/revert AS-induced HF. Beta3 adrenergic receptor (β3AR) signaling is beneficial in several forms of HF. Here, we studied the potential beneficial effect of β3AR overexpression on AS-induced HF. Selective β3AR stimulation had a positive inotropic effect. Transgenic mice constitutively overexpressing human β3AR in the heart (c-hβ3tg) were protected from the development of HF in response to induced AS, and against cardiomyocyte mitochondrial dysfunction (fragmented mitochondria with remodeled cristae and metabolic reprogramming featuring altered substrate use). Similar beneficial effects were observed in wild-type mice inoculated with adeno-associated virus (AAV9) inducing cardiac-specific overexpression of human β3AR before AS induction. Moreover, AAV9-hβ3AR injection into wild-type mice at late disease stages, when cardiac hypertrophy and metabolic reprogramming are already advanced, reversed the HF phenotype and restored balanced mitochondrial dynamics, demonstrating the potential of gene-therapy-mediated β3AR overexpression in AS. Mice with cardiac specific ablation of *Yme1l* (cYKO), characterized by fragmented mitochondria, showed an increased mortality upon AS challenge. AAV9-hβ3AR injection in these mice before AS induction reverted the fragmented mitochondria phenotype and rescued them from death. In conclusion, our results step out that β3AR overexpression might have translational potential as a therapeutic strategy in AS–induced HF.

**Supplementary Information:**

The online version contains supplementary material available at 10.1007/s00395-022-00966-z.

## Introduction

Aortic stenosis (AS) involves progressive pressure afterload in the left ventricle (LV), leading to increased mass and concomitant myocardial fibrosis [7]. In early disease stages, the LV is able to cope with the increased afterload and maintain an adequate cardiac output by increasing its contractile capacity, partly because the hypertrophy of preexisting cardiomyocytes results in global LV hypertrophy (LVH). However, as the disease progresses, the LV begins to dilate and lose contractile capacity, and the disease is characterized by heart failure (HF), in the form of symptomatic lung congestion or reduced LV ejection fraction (LVEF). HF is a strong predictor of poor prognosis in AS [48]. Untreated AS with superimposed HF is associated with a very high short-term mortality [23]. Aortic valve replacement is widely used worldwide, and available noninvasive approaches are associated with very low procedure-related complications [8]. However, aortic valve replacement often fails to reverse the preexisting myocardial dysfunction, and therefore the HF persists, together with the associated poor clinical prognosis. There is a lack of therapeutic strategies able to prevent or reverse aortic-stenosis–induced LVH and HF.

The cornerstone of therapy for many forms of HF is treatment with modulators of β1 and β2 adrenergic receptors (βAR) (beta-blockers) [41, 51]. βARs are widely distributed in the cardiovascular system, and the β1AR and β2AR variants were long thought to be the only types of these receptors, with β1AR the more highly expressed in cardiomyocytes. Much later, a third subtype (β3AR) was discovered in adipocytes, where they were shown to be involved in cell metabolism [19]. β3AR has since been detected in cardiomyocytes of different species, including humans [22]. Although cardiac β3AR levels are low in physiological conditions [40], β3AR signaling seems to play a beneficial role in myocardial conditions such as acute myocardial infarction [1, 4, 12, 21, 28, 50, 68, 75], and β3AR expression is upregulated in the hearts of HF patients [46]. While there is some debate, the weight of the evidence suggests that β3AR upregulation in HF is a beneficial defense mechanism that could be exploited therapeutically [20, 46, 47, 76].

A well-known characteristic of HF is altered mitochondrial dynamics, and this phenomenon has attracted attention as a possible target for the prevention or treatment of this condition [72]. β3AR signaling has been linked to mitochondrial homeostasis and is involved in mitochondrial biogenesis in adipocytes [5], where its stimulation significantly increases mitochondrial density during white-to-beige adipocyte reprogramming [65].

We hypothesized that β3AR overexpression might have translational potential as a therapeutic strategy in AS–induced HF.

## Methods

The investigation conforms to the Guide for the Care and Use of Laboratory Animals published by the US National Institutes of Health (NIH Publication No. 85–23, revised 1985).

### Mice

All experimental and other scientific procedures with animals conformed to EU Directive 2010/63EU and Recommendation 2007/526/EC, enforced in Spanish law under Real Decreto 53/2013. Animal protocols were approved by the local ethics committees and the Animal Protection Area of the Comunidad Autónoma de Madrid. C57BL/6 J wild-type (WT) mice and the following transgenic lines on the C57BL/6 J genetic background were used in this study, and all experiments were conducted with adult males.

### *R26-ADRB3* transgenic mice

To target the human β3AR transgene (*ADRB3*) into the *Rosa26* (*R26)* locus in embryonic stem (ES) cells by homologous recombination, we generated a construct with a *PGK-Neo* cassette plus a transcriptional stop site flanked by loxP elements followed by *ADRB3* cDNA and an *IRES-EGFP* construct, using a previously described strategy [61]. Upon Cre recombination, transgene expression will be controlled by the *R26* promoter. Complete human *ADRB3* cDNA was obtained from clone IMAGE. The sequence was PCR amplified with Phusion High-Fidelity DNA Polymerase (NEB) and primers containing *EcoR*I sites and was cloned into the *EcoR*I site of a *pCDNA3.1-IRES-EGFP* plasmid previously generated by cloning a *Sal*I *IRES-EGFP* fragment into the *Xho*I site of *pCDNA3.1*. The resulting plasmid was digested with *Xba*I*,* treated with DNA Polymerase I, large Klenow fragment (NEB) to form blunt ends and digested with *Nhe*I to obtain a *Nhe*I-blunted *Xba*I *ADRB3-IRES-EGFP* fragment. The fragment was cloned into *Nhe*I-blunted *Not*I sites of pBigT. We obtained the *Pac*I*-Asc*I-cassette containing loxP-*PGK-Neo-STOP*-loxP-*ADRB3-IRES-EGFP* by digestion and cloned it into the *Pac*I*-Asc*I sites of modified *pROSA26-*1 plasmid. The final construct was linearized with *Xho*I and electroporated into G4 ES cells derived from a 129S6/SvEvTac x C57BL/6Ncr cross [71]. After G418 (200 µg/mL) selection for 7 days, 192 clones were picked. Homologous recombination was identified by Southern blot of DNA digested with *EcoR*V and hybridized with 5' and 3' probes. Four clones were positive, and we selected two to confirm karyotype. One clone was injected into B6CRL blastocysts to generate chimeras, which were analyzed for germ line transmission. The resulting human β3AR transgenic mouse line (*ADRB3*^*tg/tg*^) was crossed with C57BL/6 J mice to achieve a pure genetic background.

### Transgenic mouse lines expressing human β3AR

Upon Cre recombination, the stop codon between the *R26* promoter and the *ADRB3*-IRES-*EGFP* sequence is removed, and the *R26* promoter drives *ADRB3* expression. We used the *cTnT*^*Cre/*+^ line, with cardiomyocyte-specific expression of Cre recombinase [34], to drive hβ3AR expression. Crossing of both lines generated c-hβ3tg mice (*cTnT*^*Cre/*+^*;ADRB3*^*tg/tg*^) with cardiomyocyte-specific overexpression of β3AR (human β3AR expressed against a background of endogenous mouse β3AR expression). Controls were wild-type (WT) littermates (*cTnT*^+*/*+^*;ADRB3*^*tg/tg*^), with normal levels of mouse β3AR.

c-hβ3tg mice were further crossbred with a knockout line with targeted disruption of the mouse β3AR gene (*adrb3*),[69] generating c-hβ3tg mβ3KO mice (*cTnT*^*Cre/*+^*;ADRB3*^*tg/tg*^*;adrb3*^*−/−*^), with sole expression of human β3AR in cardiomyocytes. Controls were mβ3KO littermates (*cTnT*^+*/*+^*;ADRB3*^*tg/tg*^;*adrb3*^*−/−*^), with no β3AR expression.

### Binding assay

Snap frozen hearts were crushed and200-400 mg of tissue was mixed with 50 mM Tris–HCl (pH 7.5). The samples were homogenized using an ultrasonic cell disruptor (MicrosonTM ultrasonic cell disruptor) keeping the sample on ice and they were filtered using nylon mesh. Next, the samples were centrifuged at 4 °C for 15 min at 1000 g. Protein in the resulting supernatant was quantified using the Bradford method and 2 mg of protein was incubated in duplicate for 60 min at 37 °C with different concentrations of [3H]-CGP 12,177 (from 0.25 to 120 nM) (Perkin Elmer, Waltham, MA, USA) in 50 mM Tris–HCl (pH 7.5). Experiments were terminated by rapid filtration through fiberglass filters (Schleicher and Schuell, GF 52), presoaked in 0.3% polyethyleneimine, using a Brandel cell harvester (M24R). The filters were then washed three times with 4 ml of ice-cold 50 mM Tris–HCl buffer (pH 7.5), and the filter-bound radioactivity was determined by liquid scintillation counting (2480 WIZARD, PerkinElmer, Waltham, Massachusetts, USA). Nonspecific binding was measured in the presence of 1 mM propranolol (Sigma). Specific binding is defined as total binding minus nonspecific binding. The saturation data were analyzed by non-linear regression using Prism version 4.0 (GraphPad Software; San Diego, California, U.S.A) to determine the maximum number of binding sites (Bmax) expressed as fmol/mg of protein.

### Western blot

Cells and tissue samples (0.1 mg) were lysed in RIPA buffer containing protease inhibitors (complete-Roche, Indianapolis, IN, USA) and phosphatase inhibitors (PhosSTOP-Roche, Indianapolis, IN, USA). The supernatant was separated by centrifugation at 12000 g for 15 min at 4 °C, and total protein concentration was detected with the BCA protein assay kit (Thermo Fisher, USA) using bovine serum albumin (BSA) as the standard. Equal amounts of protein (15ug) were separated by SDS-PAGE and transferred to a nitrocellulose membrane using a transfer apparatus according to the manufacturer’s protocol (BioRad). After incubation with 5% of nonfat milk or BSA in TBST for 60 min, membranes were incubated overnight at 4 °C with primary antibodies against GFP (1:1000; Living Colors® Full-Length GFP Polyclonal Antibody, 632,592, Clontech), OPA1 (1:1000; Thermo Fisher, PA1-16,991), MFN2 (1:1000; Abcam, ab56889), Vinc (1:1000, Sigma, V4505), UCP2 (1:2000, Genetex, GTX132072) and GAPDH (1:10,000; Abcam, ab8245). Membranes were washed 3 times for 5 min each with TBST and incubated for 1 h with HRP-conjugated anti-mouse or anti-rabbit antibodies (1:5000). Bound antibody signals were developed with the ECL (Luminata) system. Quantitative densitometry analysis was performed using Fiji (ImageJ) software.

### Histology and immunofluorescence

Heart specimens were fixed in 4% formaldehyde, dehydrated to xylene, and embedded in paraffin. After deparaffinization and rehydration, 5-μm sections were cut at 3 levels, mounted on glass slides, and stained with hematoxylin and eosin and with 1% Sirius red in picric acid (Sigma-Aldrich) to detect interstitial fibrosis. All sections were examined with a Nikon Eclipse Ni microscope and scanned with a NanoZoomer-RS scanner (Hammamatsu), and images were exported with NDP.view2. The percentage of fibrosis was quantified using Fiji (ImageJ) software in at least three sections per heart, and the mean was used for statistical analysis.

For immunofluorescence, hearts were fixed in 4% formaldehyde, dehydrated through 15% sucrose in PBS and then 30% sucrose overnight at 4 °C, and embedded in Tissue-Tek® OCT compound (SAKURA, Netherlands). Cryostat sections were blocked and permeabilized for 1 h at RT in PBS containing 0.3% Triton X-100 (90,002-93-1, Sigma), 5% BSA (A7906, Sigma), and 5% normal goat serum (055-000-001, Jackson InmunoResearch). Sections were then incubated overnight at 4 °C with anti-GFP (Living Colors® Full-Length GFP Polyclonal Antibody, 632,592, Clontech) diluted (1:500) in PBS containing 0.3% Triton X-100 and 2.5% normal goat serum. After washes, samples were incubated for 2 h at RT with a secondary antibody (Alexa Fluor, Invitrogen) and the nucleic acid stain Hoechst 33,342 (B2261, Sigma) and were mounted in Fluoromount G imaging medium (4958–02, Affymetrix eBioscience).

For cell immunofluorescence, adult mouse ventricular myocytes (AMVM) and neonatal rat ventricular myocytes (NRVM) were fixed with 4% paraformaldehyde in PBS for 10 min. Cells were then washed 1–3 times with PBS and blocked with 2% BSA (in PBS) for 1 h at RT. Samples were incubated overnight at 4 °C with primary antibodies (anti hβ3AR; A4854 Sigma and anti α-actinin; A7811 Sigma). After 1–3 washes with PBS, cells were incubated with Alexa Fluor secondary antibodies for 1 h at RT. Cells were then washed 1–3 times with PBS and incubated for 5 min with DAPI (1:10,000 in PBS) and washed again 1–3 times with PBS. AMVMs were additionally incubated with FITC-conjugated lectin for 1 h (L4895, Sigma) before a final wash in PBS. Stained cells were mounted in Fluoromount G imaging medium (4958–02, Affymetrix eBioscience).

### Mouse left ventricular catheterization and pressure–volume loops

Ventricular catheterization was performed as previously described [54]. Mice were anesthetized (sevoflurane 1.5%) and intubated. A skin incision was made to visualize the diaphragm, which was heat cauterized to expose the heart apex. The pericardium was removed gently with forceps. Using a 25–30 gauge needle, a stab wound was made near the heart apex into the left ventricle (LV). The catheter tip (Transonic, NY, USA) was inserted retrogradely into the LV until the proximal electrode was just inside the ventricular wall. The catheter position was adjusted to obtain rectangular shaped pressure–volume (PV) loops. After allowing the signal to stabilize for 5 min, recordings were made of baseline PV loops, heart rate, maximal derivative of LV pressure (dP/dtmax), minimal derivative of LV pressure (dP/dtmin), left ventricular end-systolic pressure (LVESP), minimal derivative of LV pressure (dP/dtmin), and time constant of isovolumic relaxation (Tau). The same parameters were recorded after the injection a single dose of mirabegron (1 µg/kg) through the femoral vein. At the conclusion of the experiment, the catheter was removed by gently pulling it back through the stab wound, and the animal was euthanized.

### Adeno-associated virus production and in vivo delivery

HEK293T cells were transfected using linear polyethylenimine hydrochloride with two plasmids: pDG-9 or pDG-6 plus an AAV transfer plasmid in which the transgene (*ADRB3* or *EGFP*) is placed between 2 ITRs. pDG-9 encodes Rep78, Rep68, Rep52, and Rep40 (required for the AAV life cycle); serotype 9 VP1, VP2, and VP3 (capsid proteins); and adenoviral genes E4, E2a and VA (mediating AAV replication) and was used to generate AVV-9. Similarly, pDG-6 encodes the same genes except that VP1, VP2, and VP3 are from serotype 6, and this plasmid was used to generate AVV-6. An AAV transfer plasmid encoding *ADRB3* under the control of the troponin T promoter was used to generate recombinant AAV (rAAV) for hβ3AR cardiac-specific expression (Fig. S5A). An AAV transfer plasmid encoding *EGFP* instead of *ADRB3* as used to generate control rAAV (Fig. S5B). The AAV transfer plasmids also contained an IRES sequence followed by the luciferase gene after the transgene sequence (*ADRB3* or *EGFP*). Luciferase activity was used as a reporter to check viral transduction in vivo.

Cells were harvested 3 days after transfection, lysed, frozen and thawed three times, and digested with benzonase (150 units/mL). The final supernatant containing the virus was then purified on an iodixanol gradient in an optiseal polypropylene tube (361,625, Beckman Coulter). The concentrated and purified viral fraction was collected between the 40% and 60% iodixanol layers after ultracentrifugation (350,333 g, 18 °C for 1 h).

Adult mice were anesthetized and maintained on 1–2% isoflurane. A skin incision was made on the medial face of the hindlimb, and the femoral vein was exposed. A dose of 3 × 10^11^ viral genomes (vg)/mouse in 50 µl saline was injected using a 31G insulin syringe, and the skin was closed with a 6/0 silk thread. AAV-9 was used for in vivo delivery.

### In vivo and ex vivo imaging system for luminescence detection

To verify correct viral transfection, 875 µg of D-luciferin (Xenogen, Alameda, CA) was administered to mice in a volume of 50 µl by intraperitoneal injection. Three minutes later, animals were anesthetized and maintained on 1–1.2% isoflurane in oxygen. Six minutes after D-luciferin administration, all mice were imaged using a Xenogen IVIS100 imaging system. Emitted photons were collected and integrated over 2 min periods. Images were processed using Xenogen Living Image software. For ex vivo bioluminescence imaging, animals were killed, organs were removed and quickly dipped in D-luciferin (17.5 g/mL), and images were captured with a supercooled charge-coupled camera. Emitted photons were collected and integrated over 2 min periods. Results are expressed as mean luminescence intensities (photons/s/cm2/sr).

### RNA extraction and cDNA preparation

Tissues were homogenized using TissueLyser (Qiagen), and total RNA was extracted with QIAzol reagent (Qiagen). The RNA pellet was dissolved in RNase-free water, and concentration was measured in a NanoDrop spectrophotometer (Wilmington). RNA (2 µg) was transcribed to cDNA using the High Capacity cDNA Reverse Transcription Kit (Applied Biosystems).

### ADRBs PCR

cDNA (100 ng) was amplified by PCR using DNA polymerase (Biotools, Spain). PCR products were separated on a 2% agarose gel containing ethidium bromide. Images were taken with a Molecular Imager® Gel Doc™ XR + System (BioRad). Primers were designed specifically to match only the human *ADRB3* cDNA sequence and not the mouse sequence: (Forward primer: TGCCAATTCTGCCTTCAACC; Reverse primer: CAGGCCTAAGAAACTCCCCA). Primers used to evaluate endogenous mouse *Adrb1* and *Adrb2* transcript levels in the context of transgenic *Adrb3*overexpression were: *Adrb1*(Forward primer: TCATCGTGGTGGGTAACGTG; Reverse primer: ACCAGCAATCCCATGACCAG); *Adrb2*(Forward primer: TTCGAAAACCTATGGGAACG; Reverse primer: GGGATCCTCACACAGCAGTT). To compare the expression level of the hβ3AR compared to the endogenous m *Adrb3* levels, we used the following primers: mouse *Adrb3* (Forward primer: TGATGGCTATGAAGGTGCG; Reverse primer: AAAATCCCCAGAAGTCCTGC); human *Adrb3*(Forward primer: TGCCAATTCTTGCCTTCAACC; Reverse primer: CAGGCCTAAGAAACTCCCCA).

### Adult mouse ventricular myocyte isolation

The protocol for AMVM isolation was as previously described [21]. Briefly, 10- to 12-week-old mice were heparinized (50 USP units) and anesthetized with a mixture of ketamine (140 mg/kg), xylazine (33 mg/kg), and atropine (9 mg/kg). Once pedal pinch reflexes were completely inhibited, animals were placed in a supine position, ventral thoracic regions were wiped with 70% alcohol, and animals were euthanized. The heart was quickly removed, cannulated through the ascending aorta, and mounted on a modified Langendorff perfusion apparatus. The heart was then retrogradely perfused (3 mL/min) for 5 min at RT with prefiltered Ca^2+^-free perfusion buffer (113 mM NaCl, 4.7 mM KCl, 0.6 mM KH_2_PO_4_, 0.6 mM Na_2_HPO_4_, 1.2 mM MgSO_4_-7H_2_O, 12 mM NaHCO_3_, 10 mM KHCO_3_, 0.032 mM Phenol Red, 0.922 mM Na-HEPES, 30 mM taurine, 5.5 mM glucose, and10 mM 2,3-butanedione-monoxime; pH 7.4). Enzyme digestion was performed for 20 min at 37ºC in digestion buffer [perfusion buffer containing 0.2 mg/mL Liberase™, 2.5% (5.5 mM) trypsin, 5 × 10^–3^ U/mL DNase, and 12.5 µM CaCl_2_]. At the end of the enzyme digestion, both ventricles were isolated and gently disaggregated in 5 mL digestion buffer. The resulting cell suspension was filtered through a 100 µm sterile mesh (SEFAR-Nitex) and transferred to a tube containing 10 mL stopping buffer-1 [perfusion buffer supplemented with 10% v/v fetal bovine serum (FBS) and 12.5 µM CaCl_2_]. After gravity sedimentation for 20 min, cardiomyocytes were resuspended in stopping buffer-2 (as stopping buffer-1 but with 5% v/v FBS) for another 20 min. Cardiomyocytes were reloaded with Ca^2+^ by 10 min incubations in stopping buffer-2 with five progressively increasing CaCl_2_ concentrations (62 µM, 112 µM, 212 µM, 500 µM, and 1 mM). Resuspension and decanting of cells at each step contributed to the purification of the cardiomyocyte suspension. The homogeneous suspension of rod-shaped cardiomyocytes was then resuspended in M199 supplemented with Earle’s salts and L-glutamine (5 mM), 1% penicillin–streptomycin (P/S), 0.1 × insulin–transferin–selenium-A, 2 g/L BSA, 25 µM blebbistatin, and 5% FBS. Cells were plated in single drops onto 22 mm^2^ glass coverslips precoated with 200 µL mouse laminin (10 mg/mL) in PBS for 1 h.

### Adult mouse hearts perfusion

The protocol for mouse adult ventricular myocytes is described here [21]. Briefly, 10- to 12-week-old mice were euthanized with CO2. Once pedal pinch reflexes were completely inhibited, animals were placed in a supine position and ventral thoracic regions were wiped with 70% of ethanol. The heart was quickly removed, cannulated through ascending aorta, and mounted on a modified Langendorff perfusion apparatus. The heart was retrogradely perfused for 20 min at room temperature with Perfusion Buffer [NaCl (113 mmol/L); KCl (4.7 mmol/L); KH2PO4 (0.6 mmol/L); Na2HPO4 (0.6 mmol/L); MgSO4-7H2O (1.2 mmol/L); NaHCO3 (12 mmol/L); KHCO3 (10 mmol/L); Phenol Red (0.032 mmol/L); HEPES-Na Salt (0.922 mmol/L); taurine (30 mmol/L); glucose (5.5 mmol/L); 2,3-butanodione monoxime (10 mmol/L), pH 7.4]. Perfusion Buffer was supplemented with 3.8 × 10–9 M of Mirabegron (SML2480-Merck) and 0.45 mM of IBMX (P019-Quimigen). Heart was stored at -80ºC for processing.

### cNMP quantification

Cyclic AMP and GMP levels were measured in ether-extracted samples by EIA using cAMP (501,040) and cGMP (581,021) kits from Cayman Chemical, performed according to the manufacturer’s instructions. Both cyclic AMP and GMP levels (pM) were then normalized by μg of heart tissue protein.

### ATP quantification

ATP levels were measured in heart samples using an EnzyLight™ ATP Assay Kit (EATP-100, BioAssay Systems), performed according to the manufacturer’s instructions. ATP concentrations (μM) were then normalized by μg of heart tissue protein.

### Neonatal rat ventricular myocyte isolation

The protocol for hypoxia/reoxygenation in NRVMs was as described previously [13]. Ventricular cardiomyocytes were isolated from 1- to 2-day-old neonatal rat hearts. Hearts were prewashed in ADS buffer (116 mM NaCl, 20 mM HEPES, 0.8 mM Na_2_HPO_4_, 5.6 mM glucose, 7 mM KCl, and 0.8 mM MgSO_4_·7H_2_O; pH 7.35) to remove blood and then placed in dishes containing 7 mL ADS. Hearts were then minced into small pieces with sterile razor blades, and the tissue suspensions were then transferred to flasks containing 7 mL of enzyme solution (ADS containing 0.6 mg /mL pancreatin, 8820 U /L collagenase II, and 50 mM CaCl_2_) and incubated for 10 min at 37 °C. The supernatant from this predigestion step was discarded, and the tissue pieces were incubated in 15 mL of digestion solution for 15-min periods at 37 °C. At the end of each incubation period, the supernatant was collected in 50 mL conical tubes containing 19 mL F-10 medium and 20% FBS preheated to 37 °C. Three-six fractions were collected and centrifuged at 1400* g* for 10 min, the supernatant was discarded, and cells in each tube were washed with 5 mL FBS. The cells were then centrifuged at 1400* g* for 10 min, and the supernatant was discarded. The resulting pellet containing NRVMs was resuspendend in HAM’s F10 complete medium supplemented with 10% horse serum (HS), 5% FBS, and 1% P/S, pH 7.4. The cell suspension was filtered through a 70 μm filter and preplated for 2 h on a Nunc Nunclon 100 mm cell culture dish (Thermo Fisher Scientific, Waltham, MA, USA) to separate fibroblasts from the myocyte fraction. The supernatant, containing mostly myocytes, was collected and plated on culture dishes in Hams F-10 complete medium. The fibroblasts attached to the Nunclon dishes were cultured in DMEM containing 1% P/S. Cells were maintained in medium without HS and supplemented only with 10% FBS and 1% P/S. Experimental treatments and controls were conducted in serum-free medium.

### Neonatal rat ventricular myocyte transfection

Recombinant AAV6 were generated encoding the hβ3AR gene under the control of a truncated chicken cardiac troponin-T (cTnT) promoter and strengthened by the Cmr4 enhancer. The luciferase reporter gene placed after the hβ3AR gene was used to confirm expression and track transduced cells. The β3AR and luciferase genes were separated by an IRES sequence to prevent formation of a fusion protein that could alter β3AR function. A polyA sequence was added at the end to confer mRNA stability. The gene construct was flanked by ITR sequences for AAV machinery recognition. NRVMs were isolated and cultured for 24 h before being transfected with recombinant AAV-6 at the MOI indicated in each figure. A 10 K MOI was used in NRVM hypertrophy and chronotropy experiments. Transfections were performed in free-serum medium for 12 h, Ham’s F-10 complete medium was added, and transgene expression was allowed for 72 h before experiments.

The efficiency of AAV6 transduction of NRVMs was checked at 48 h and 72 h by monitoring luciferase activity in cells transduced with the AAV6-EGFP control virus. Luciferase reporter luminescence identified a 10 K MOI as the appropriate dose for subsequent experiments.

### Neonatal rat ventricular myocyte luciferase assay

NRVMs (2 × 10^5^ cells per well in a 24-well multi-well dish) were transduced with AAV-6 containing the luciferase gene. NRVMs were washed three times with ice cold PBS and collected in passive lysis buffer (Promega, Madison, WI, USA). Luciferase activity was measured with a luciferase assay system kit (Promega) and a plate reader (Infinite M1000 PRO-TECAN).

### Neonatal rat ventricular myocyte hypertrophy

The protocol was as previously described [13]. After isolation, NRVMs (~ 3 × 10^5^ cells per well) were plated in a six-well multi-well dish, transfected for 72 h with AAV6, and stimulated as indicated (isoproterenol, 10 μM; L-NAME, 100 μM; or both). NRVMs were then fixed in 3% PFA for 10 min, washed three times with ice cold PBS, and permeabilized with 0.2% Triton X-100. The cells were then incubated with 1% BSA for 30 min and incubated overnight at 4 °C with anti-α-sarcomeric actinin (α-SMA, A7811, Sigma-Aldrich) diluted 1:200 in 1% BSA. After washes, cells were incubated with FITC-conjugated secondary antibody anti-mouse (Sigma-Aldrich; 1:200). Cells were examined with a Nikon Eclipse Ni microscope, and images were acquired with a Nikon digital camera. For each sample, five to six fields (~ 50 cells per field) were acquired.

### Mouse model of supravalvular aortic stenosis (AS) by transaortic constriction (TAC)

Male 8- to 12-week-old mice were intraperitoneally anesthetized with ketamine (60 mg/ kg), xylacine (20 mg/kg), and atropine (9 mg/kg). Once deeply asleep, animals were orally intubated under direct tracheal visualization using a blunted 22G cannula, and mechanical ventilation was maintained throughout the procedure (SAR-830. CWE Inc).

While models of valvular AS have been recently refined [62], we decided to use the more widely described model of supravalvular AS by TAC [64], since it resembles many of the features of AS-induced HF [29]. Partial thoracotomy to the second rib was performed under a surgical microscope, and the sternum was retracted with a chest retractor. Fine tip 45° angled forceps were used to gently separate the thymus and fat tissue from the aortic arch. After identification of the transverse aorta, a small piece of a 7.0 prolene suture was placed between the brachiocephalic and left carotid arteries. Two loose knots were tied around the transverse aorta, and a small piece of a blunt 27 gauge needle was placed parallel to the transverse aorta. The knots were quickly tied against the needle, and the needle was removed, leaving 0.36 mm diameter constriction. In sham-operated control mice, the entire procedure was identical except that the aortic ligation of the aorta was omitted. The chest retractor was removed, and the ventilator outflow was pinched off for 2 s to re-inflate the lungs. The rib cage was closed with a 6.0 silk suture using an interrupted suture pattern. The skin was closed with a 6.0 silk suture using a continuous suture pattern. Animals were allowed to recover in a warmed cage with a 98% oxygen supply.

### Echocardiography

Echocardiographic evaluations of mice were performed by an experienced observer blinded to the study at baseline and at 1, 3, 4, 5, 8, 10, and 12 weeks post-TAC, depending on the experiment. Mice were lightly anesthetized with 0.5–2% isoflurane in oxygen, administered via a nose cone and isoflurane delivery adjusted to maintain a heart rate of 450 ± 50 bpm. Anesthetized mice were placed in a supine position on a heated platform, and warmed ultrasound gel was used to maintain normothermia. Mice were examined with a 30-MHz transthoracic echocardiography probe and a Vevo 2100 ultrasound system (VisualSonics, Toronto, Canada). A base-apex electrocardiogram (ECG) was continuously monitored through 4 leads placed on the platform and connected to the ultrasound machine. Images were transferred to a computer and were analyzed off-line using the Vevo 2100 Workstation software. For the assessment of LV systolic function, standard 2D parasternal long axis views were acquired at a frame rate > 230 frames/sec. End-systolic and end-diastolic LV volumes (LVESV and LVEDV) and LV ejection fraction (LVEF) were calculated using the area-length method. LV mass was calculated from short-axis M-mode views using end-diastolic left ventricular wall thickness.

### In vivo treatment with L-NAME

Wild-type and c-hβ3tg male 8- to 12-week-old mice were treated for 15 days with the nitric oxide synthase inhibitor L-NAME (1 mg/mL). Systolic and diastolic arterial pressures were measured after 2-week of L-NAME treatment. Afterwards, mice were subjected to TAC surgery and followed up to monitor survival.

### Lung water content

Lungs were first weighed and then dried for 7 days in a 60 °C oven. The mass of the dry lung was then measured, and water content was calculated as the difference in mass between the dry and wet lung, expressed as a percentage.

### Positron emission tomography – computed tomography

All PET-CT studies were performed with a small-animal PET-CT device as previously described [72]. Briefly, animals were fasted overnight, and anatomic thorax CT scans were performed 1 h after [^18^F]FDG injections, followed by metabolic PET static acquisition for 15 min. Prefused and prereconstructed images were analyzed with Osirix (Aycam Medical Systems, LLC); we selected myocardium of the whole heart and calculated the mean myocardial standardized uptake value (SUV med) for each animal.

### Transmission electron microscopy

Transmission electron microscopy (TEM) was performed as previously described [16]. Immediately after excision, LV samples were fixed in 4% formahaldehyde: 1% glutaraldehyde in cacodylate buffer, and postfixed in 1% osmium tetroxide. Tissues were then washed in PBS, dehydrated through graded alcohols followed by acetone, and then infiltrated with Durcupan ACM Fluka resin and polymerized at 60 °C for 48 h. Blocks were cut with a Leica ultracut UCT ultramicrotome (Leica, Heerbrugg, Switzerland), and Sects. (60–70 nm) were mounted onto 200-mesh grids. Sections were stained with a 2% solution of aqueous uranyl acetate for 10 min, followed by lead citrate staining for 10 min. Stained sections were viewed with a JEOL JEM-1010 transmission electron microscope (Tokyo, Japan) operating at 80 kV through 6000 × , 10,000 × , and 40,000 × objectives. Images were acquired with a GATAN Orius 200SC digital camera. Mitochondrial morphometry was analyzed using ImageJ (National Institutes of Health).

### Seahorse

The bioenergetic response of AMVMs was measured with the Seahorse Bioscience XF96 Flux Analyzer, as previously described [72]. For glucose and palmitate tolerance experiments, cells were preincubated for 30 min in 160uL unbuffered DMEM supplemented with 4 mM glutamine, 1 mM pyruvate, 5 mM glucose, 0.5 mM L-carnitine, and BSA (0.17 mM)-conjugated palmitate (0.4 mM) for 30 min. The XF96 automated protocol consisted of a 10 min delay after microplate insertion, baseline OCR/ECAR measurements [3x (3 min mix, 3 min measure)], followed by injection of port A (20uL) containing the β3AR agonist BRL37344 (1 μM), and OCR/ECAR measurement [3x (3 min mix, 3 min measure)]. PortB, PortC, and Port D were injected and measured similarly to Port A. Final concentrations of glucose (10, 20, and 40 mM) were adapted to the final volume increase: 200uL after Port B injection, 220uL after Port C, and 240 after Port D. Final concentrations for palmitate characterization were 0.03 mM after Port B, 0.3 mM after Port C, and 3 mM after Port D. All values were first normalized to protein content in each well and then normalized to baseline values in order to compare 8 independent experiments.

### Statistics

Experimental data are presented as mean ± standard error of the mean (SEM) and were analyzed with Prism software (Graph pad, Inc.). For normally distributed variables, comparisons between two groups were made by unpaired two-tailed Student *t*-test; for nonnormally distributed variables, the nonparametric Wilcoxon-Mann–Whitney test was used. Comparisons between more than two groups were made by two-way ANOVA with Tuckey’s post hoc test. Comparisons between more than two groups in response to increasing drug dose, substrate concentration, or time exposure were made by two-way ANOVA with Sidak's multiple comparisons test. Power calculations were used to obtain statistical significance at p-values below 0.05; *p < 0.05, **p < 0.01, ***p < 0.001, ****p < 0.0001.

## Results

### Cardiomyocyte-specific human β3AR overexpression prevents cardiomyocyte hypertrophy upon catecholamine challenge and strengthens NO/cGMP pathway

Advanced stages of AS with associated HF are characterized by increased levels of circulating catecholamines. The augmented adrenergic state and increased afterload results in significant cardiomyocyte hypertrophy. HF is associated with β3AR overexpression [47], but the impact of this upregulation on cellular hypertrophy has remained unclear [6]. Starting at a cellular level, we began with a translational approach (gene-therapy–mediated overexpression) to explore this question, in which we examined the consequences of overexpressing β3AR in neonatal rat ventricular myocytes (NRVM).

**Fig. 1:**
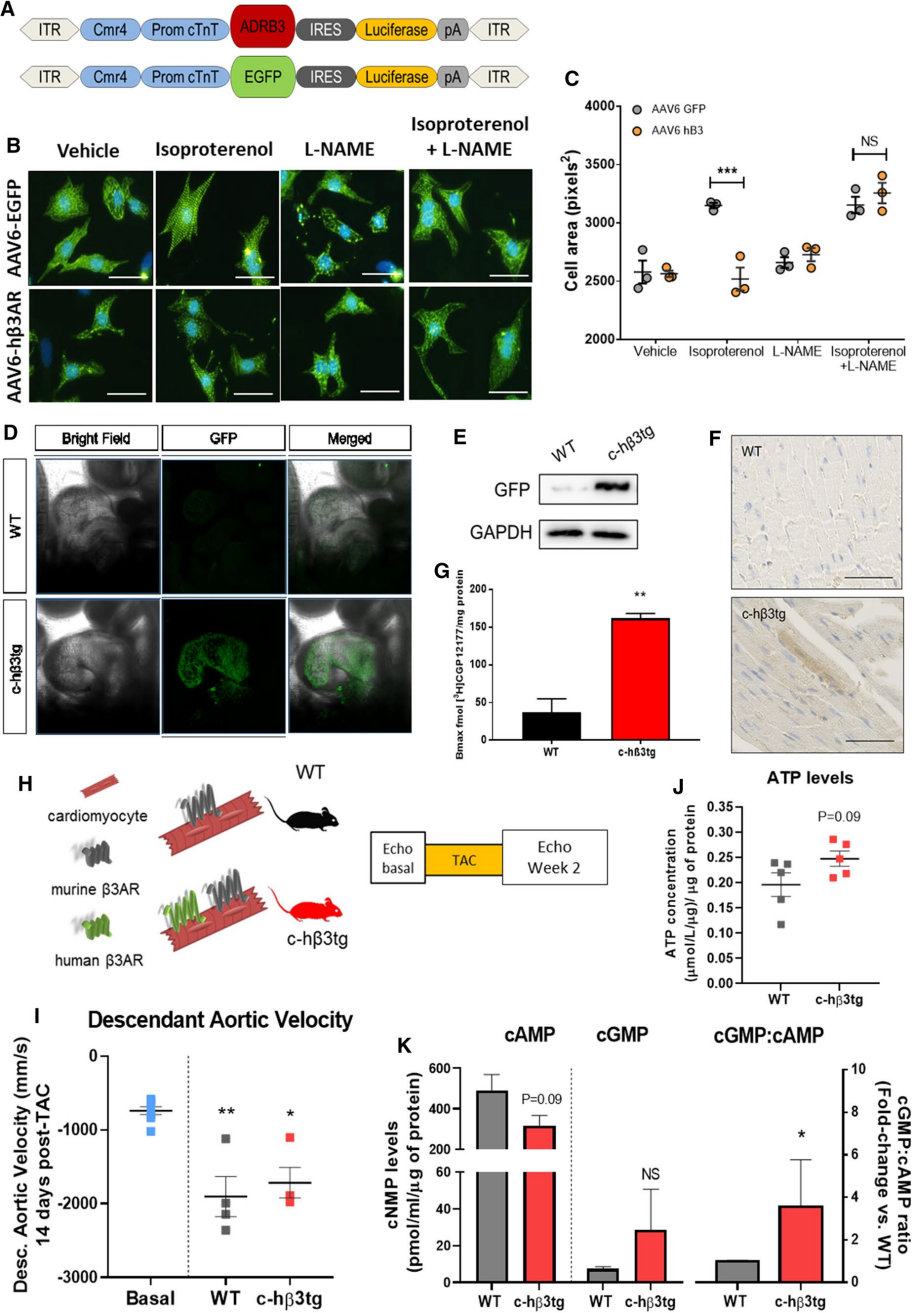
Cardiomyocyte-specific human β3AR overexpression prevents cardiomyocyte hypertrophy upon catecholamine challenge via NO/cGMP pathway. **A** Genetic constructs for adeno-associated virus (AAV) vectors encoding hβ3AR and control EGFP. ITR, recognition site for AVV packaging; Cmr4, enhancer sequence; Prom cTnT, troponin T promoter sequence for cardiomyocyte-specific expression; ADRB3, human β3AR receptor cDNA sequence, *EGFP* enhanced green fluorescent protein sequence, *IRES* internal ribosome entry site, Luciferase, firefly luciferase sequence; pA, simian virus 40 polyadenylation signal. **B** Representative images of neonatal rat ventricular myocytes (NRVM) transduced with control (AAV6-EGFP) or human β3AR adeno-associated virus (AAV6-hβ3AR) for 72 h and incubated for 24 h with isoproterenol (10 μM), L-NAME (100 μM) or both. Nucleus is stained in blue with DAPI and α-actin is stained in green to differentiate myocytes from other cells. Scale bar, 60 μm. **C** Size assessment of NVRM treated as above (40 cells/condition in each preparation; 3 independent preparations). The isoproterenol-induced hypertrophic response is blunted in hβ3AR myocytes and NOS inhibition by L-NAME restores the hypertrophy. **D** Confocal microscopy images of E9.5 *cTnT*^+*/*+^*;R26ADRB3*^*tg/tg*^ (control) and *cTnT*^*Cre/*+^*;R26ADRB3*^*tg/tg*^ (c-hβ3tg) embryos, showing cardiac expression of GFP in an E9.5 embryo. **E** Immunoblot showing GFP expression in cardiomyocytes isolated from adult c-hβ3tg mice. **F** Immunostaining analysis for GFP in cardiac tissue. Scale bar, 50 µm. **G** β3AR protein levels is increased in c-hβ3tg mice. β3AR density (Bmax) in fmol of [3H]-CGP12177 specifically bound/ mg protein in homogenates from c-hβ3tg (red, *n* = 3) and WT (black, *n* = 3) hearts. **H** Mice with cardiomyocyte-specific overexpression of human β3AR (c-hβ3tg, red) and littermate controls (WT, black) were subjected to transaortic constriction (TAC) surgery (to induce supravalvular AS) or sham surgery and were followed for 2 weeks. **I** Supravalvular AS was confirmed by echocardiography as an increase in the descendant aortic velocity blood flow. **J** ATP levels were increased in hearts from c-hβ3tg 2 weeks after supravalvular AS induction (*n* = 5/condition). **K** Cyclic GMP:AMP levels ratio was boosted in hearts from c-hβ3tg mice, thus suggesting an enhancing effect of human β3 overexpression in cardiomyocytes on NO/cGMP signaling (*n* = 5/condition). Data are means ± SEM. Mann–Whitney or Student’s *t* test for non-normally or normally distributed data, and Kruskal–Wallis test with Dunn’s multiple comparisons test. *p < 0.05, **p < 0.01, ***p < 0.001, ****p < 0.0001. NS, not significant

We first generated recombinant adeno-associated virus (AAV) serotype 6 encoding the hβ3AR gene driven by the cardiac troponin T (cTnT) promoter and the Cmr4 enhancer (Fig. 1A, Fig. S1A, S1B). NRVMs transduced for 72 h with a 10 K multiplicity of infection (MOI) of AAV6-hβ3AR (Fig. S2A, S2B) were immunostained to monitor hβ3AR protein subcellular distribution. The receptor was present in cardiomyocyte cell membranes (Fig. S2C). We next evaluated the effect of β3AR overexpression on catecholamine-induced hypertrophy. NRVMs were first transduced for 72 h and then incubated for 24 h with 10 µM isoproterenol. Cardiomyocytes were fixed and immunostained for α-actin to visualize cell shape and to differentiate them from fibroblasts, since these were primary culture experiments (Fig. 1B). As expected [6], cardiomyocyte size increased in AAV6-EGFP NRVMs after 24 h of isoproterenol incubation, whereas hβ3AR overexpression prevented catecholamine-induced cardiomyocyte hypertrophy (Fig. 1B, upper panels). This antihypertrophic effect was indicative of cardioprotective characteristics of the β3AR overexposure to catecholamines. Besides, when incubated with the NOS inhibitor L-NAME, AAV6-hβ3AR myocytes showed an increased size (Fig. 1B, lower panels). The cardioprotection afforded by β3AR overexpression was then abolished by L-NAME indicating a NO-dependent protective pathway (Fig. 1B, 1C) [6].

Because the increase in catecholamine’s production is a main feature of HF, we therefore evaluated the translational potential of β3AR overexpression in vivo. For this, we generated a transgenic mouse line with cardiac-specific overexpression of human β3AR (c-hβ3tg mice). A construct including the hβ3AR gene followed by an EGFP reporter gene was introduced into the *ROSA26* locus to generate hβ3AR transgenic mice (*ADRB3*^*tg/tg*^). c-hβ3tg mice were generated by crossing the *ADRB3*^*tg/tg*^ mice with a driver line expressing Cre under the control of the troponin T promoter (*cTnT*^*Cre/*+^). Confocal analysis of transgene expression in embryos revealed clear EGFP reporter-gene expression in the heart (Fig. 1D). Cardiac-specific EGFP expression in adult animals was demonstrated by immunoblot analysis of isolated cardiac myocytes (Fig. 1E) and immunostaining of cardiac tissue (Fig. 1F). The presence of hβ3AR protein in the hearts of adult animals was confirmed by binding assays with the radioactive ligand [^3^H]CGP12177 [2, 30] (Fig. 1G). The overexpression of hβ3AR was confirmed in our transgenic model (c-hβ3tg) by qPCR, while neither the mouse gene expression for β3AR, or β1AR and β2AR were affected (Fig. S4).

Supravalvular AS was induced in c-hβ3tg mice and their littermates (WT) in order to assess whether c-hβ3tg mice displayed higher levels of ATP and cyclic NMPs (cAMP and cGMP). Mice were monitored by echocardiography before and 2 weeks after supravalvular AS surgery (Fig. 1H). The increase in descendant aortic velocity confirmed the supravalvular AS (Fig. 1I). 2 weeks after AS-induction, ATP levels were increased in hearts from c-hβ3tg mice compared to WT littermates (Fig. 1J), which suggested an improved energetic performance. Consistent with our previous results in AAV6-hβ3AR NRVMs, cGMP:cAMP ratio was significantly increased in c-hβ3tg mice 2 weeks after supravalvular AS (Fig. 1K), which further verify a potentiation of the NO/cGMP pathway through β3AR overexpression in heart. The implication of cAMP-driven positive inotropic effect due to β3AR overexpression (Fig S3) is unclear. While the levels of cAMP were apparently not modified in hearts from c-hβ3tg mice (Fig. 1K), when these hearts were perfused ex vivo with the β3AR agonist mirabegron, an increase both in cGMP and cAMP were observed, although the former was to a much higher degree (Fig. S5).

All these promising results led us to perform in vivo experiments using a long-term pressure overload heart failure model in c-hβ3tg and WT mice.

### Cardiac-specific human β3AR overexpression protects against aortic-stenosis–induced LVH and heart failure

**Fig. 2:**
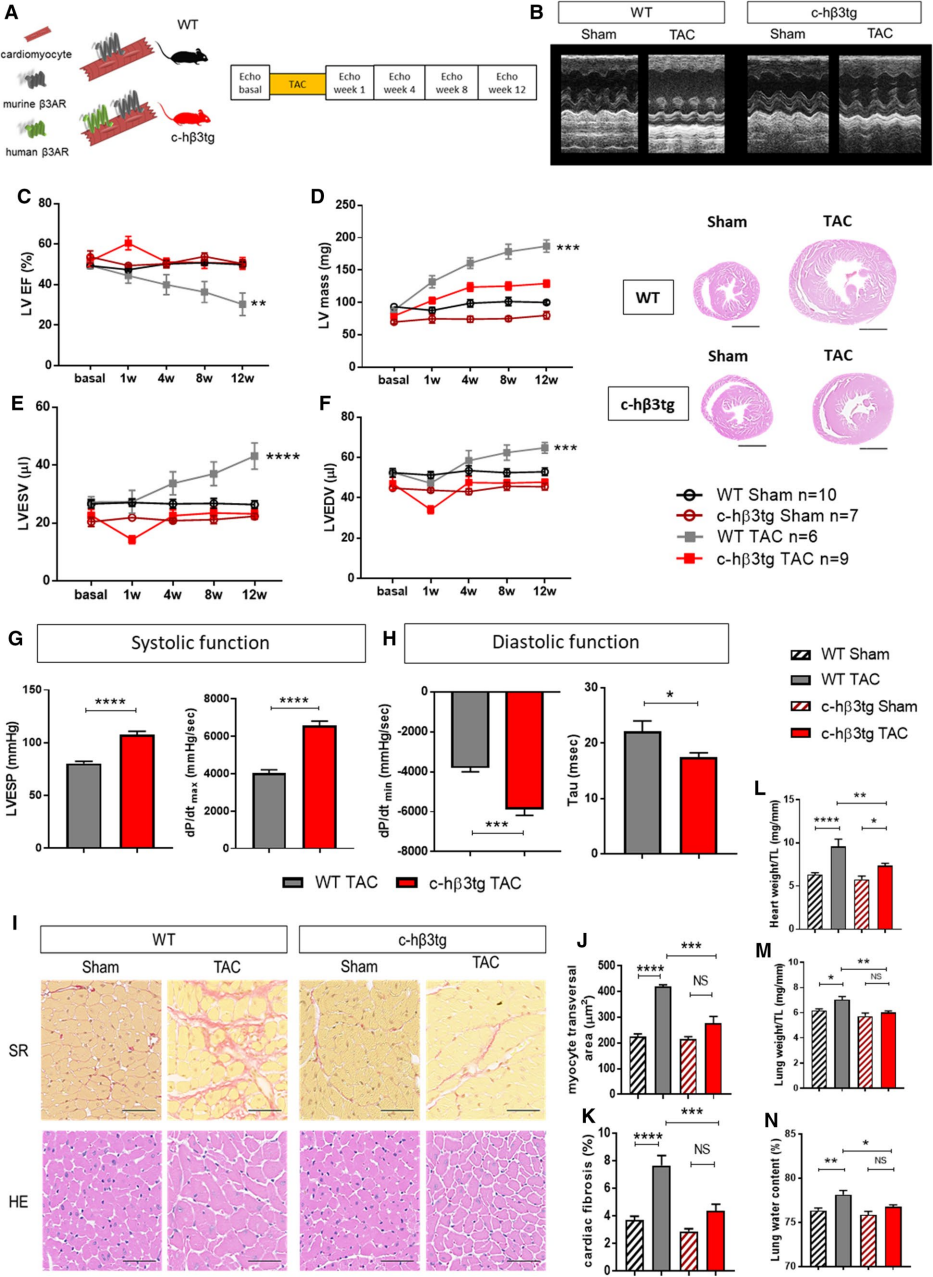
Cardiomyocyte-specific β3AR overexpression protects against cardiac hypertrophy and ventricular dilation, reduces myocyte hypertrophy and cardiac fibrosis, and preserves cardiac function. **A** Mice with cardiomyocyte-specific overexpression of human β3AR (c-hβ3tg, red) and littermate controls (WT, black) were subjected to transaortic constriction (TAC) surgery (to induce supravalvular AS) or sham surgery and were followed for 12 weeks. **B** Representative left ventricle M-mode echocardiograms (upper panels) and heart sections (lower panels) 12 weeks after surgery. Scale bar, 2 mm. **C**–**F** Echocardiographic assessment of left ventricular ejection fraction (LVEF), left ventricular mass, and left ventricular internal volume in systole (LVESV) and diastole (LVEDV). Two-way ANOVA, **p < 0.01, ***p < 0.001, ****p < 0.0001. **G** Systolic function, assessed 12 weeks after surgery by left ventricular end-systolic pressure (LVESP) and maximal derivative of LV pressure (dP/dt_max_). **H** Diastolic function, assessed 12 weeks after surgery by minimal derivative of LV pressure (LV dP/dt_min_) and the time constant of isovolumic relaxation (Tau). Data are means ± SEM. *n* = 7–8. Student *t*-test, *p < 0.05, ****p < 0.0001. **I** Fibrosis and cardiomyocyte size in c-hβ3tg and WT mice 12 weeks after TAC and sham surgery. Histological images of heart sections show staining with the fibrosis marker sirius red (SR) and hematoxylin–eosin (HE). Scale bar, 50 μm. **J** Quantification of total area of fibrosis in heart sections (*n* = 6–8). **K** Quantification of left ventricular cardiomyocyte cross sectional area (*n* = 3). **L** Heart weight normalized to tibia length (TL) (*n* = 6–8). **M** Lung weight normalized to tibia length (*n* = 6–8). N Lung water content as a percentage of fresh lung weight (*n* = 6–8). Data are means ± SEM. Two-way ANOVA with Tukey’s multiple comparisons test. *p < 0.05, **p < 0.01, ***p < 0.001, ****p < 0.0001. *NS* not significant

Supravalvular AS was induced in c-hβ3tg mice and their littermates (WT), and mice were monitored by serial echocardiography examinations over 12 weeks (Fig. 2A and B). In WT mice, AS triggered a progressive increase in cardiac mass that was severe at 12 weeks follow-up (Fig. 2D and E). Severe LVH was associated with progressive LV dilation (Fig. 2F and G) and a progressive decline in systolic cardiac function, with LV ejection fraction (LVEF) reduced to 30% at the end of the 12-week protocol (Fig. 2C). In contrast, c-hβ3tg mice showed a much milder phenotype: LV mass was also increased but to a lesser extent and there were no signs of HF, with LVEF and LV volumes maintained within normal ranges throughout the 12-week follow-up (Fig. 2C–G). LV systolic and diastolic function evaluated by invasive intracardiac pressure/volume evaluation at the end of the 12-week protocol confirmed the echocardiography results (Fig. 2H and I).

Postmortem examination revealed significantly lower heart weight in c-hβ3tg mice than in WT animals (Fig. 2B lower panel, and M). Analysis of lung weight and lung water content revealed increases in both parameters in WT mice with induced AS (Fig. 2N and O), indicating pulmonary remodeling and edema, which are key features of congestive HF. In contrast, c-hβ3tg mice showed no sign of congestive HF (Fig. 2N and O). Histological analysis of heart sections from c-hβ3tg mice revealed fewer hypertrophied cardiomyocytes (Fig. 2L) and less interstitial fibrosis (Fig. 2J and K).

15-day treatment with L-NAME in c-hβ3tg mice before TAC induction led to a significantly less survival than c-hβ3tg not receiving L-NAME (Fig. S6). This experiment points to the same direction and supports the hypothesis of cGMP/NO signaling being involved in the cardioprotection afforded by hβ3AR overexpression.

### Cardiac-specific human β3AR overexpression prevents aortic-stenosis–induced metabolic reprogramming and altered mitochondrial dynamics

**Fig. 3:**
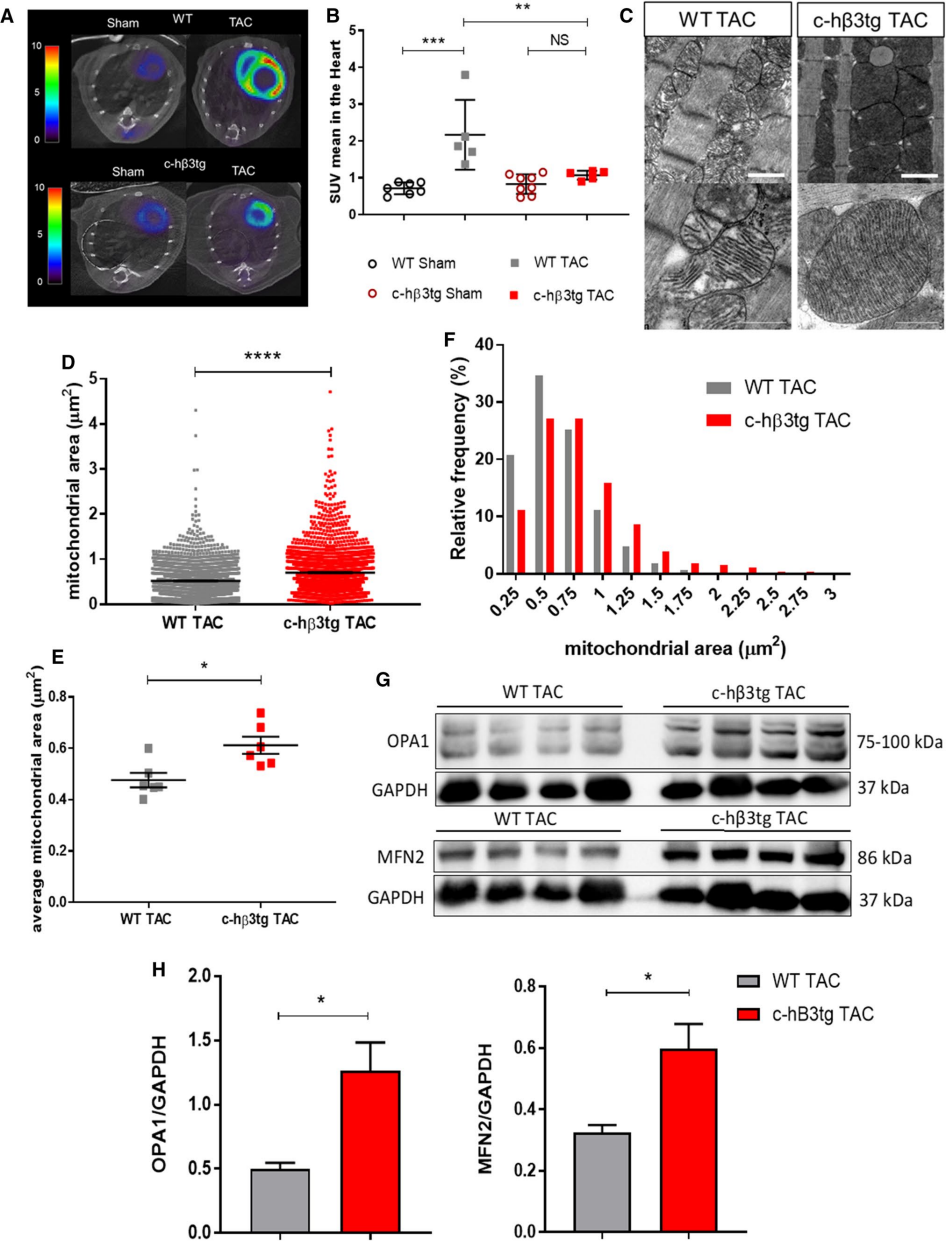
Cardiomyocyte-specific human β3AR overexpression prevents aortic-stenosis–induced metabolic reprogramming and altered mitochondrial dynamics. c-hβ3tg mice and littermate controls (WT) were subjected to TAC or sham surgery, and animals were analyzed after 12 weeks. **A** Representative thoracic PET-CT scans after [^18^F]FDG injection, as an index of glucose metabolism. B Mean standardized uptake value (SUV) for [^18^F]FDG in WT (*n* = 7) and c-hβ3tg (*n* = 8) sham-operated hearts and in WT (*n* = 5) and c-hβ3tg (*n* = 5) TAC hearts. Data are means ± SEM. Two-way ANOVA with Tukey’s multiple comparisons test. **p < 0.01, ***p < 0.001. *NS* not significant. **C** Representative transmission electron microscopy (TEM) images of heart sections from WT and c-hβ3tg mice 12 weeks after TAC (thick scale bar, 1 μm; thin scale bar, 500 nm). Note the more fragmented and damaged mitochondria (disorganized cristae) in WT hearts. **D** Mitochondrial surface area imaged by TEM in heart tissue from TAC-operated WT mice (*n* = 2233 mitochondria) and c-hβ3tg mice (*n* = 2143 mitochondria). **E** Median mitochondrial size per mouse in cardiac tissue from TAC-operated WT mice (*n* = 6) and c-hβ3tg mice (*n* = 6). F Frequency distribution of mitochondrial area in TAC-operated WT mice (*n* = 2233 mitochondria) and c-hβ3tg mice (*n* = 2143 mitochondria). Data are means ± SEM. Student *t*-test, *p < 0.05, ****p < 0.0001. **G** Western blot analysis of OPA1 and MFN2 expression in TAC-operated WT and β3tg mice. GAPDH was used for normalization of protein loading. **H** Densitometric comparison of mean OPA1 and MFN2 protein expression. Data are means ± SEM (*n* = 4). Unpaired Student *t*-test, *p < 0.05

Under physiological conditions, healthy hearts obtain energy predominantly from β-oxidation of fatty acids and a lesser proportion from glucose metabolism [72]. This situation is reversed in failing hearts: fatty-acid use is reduced, and glucose becomes the preferential substrate for energy generation [14, 39]. This metabolic switch is thought to contribute to the maintenance of HF [72]. The bias toward fatty-acid β-oxidation in c-hβ3tg cardiomyocytes (Fig. S7) therefore prompted us to explore the metabolic profile of c-hβ3tg mice with AS in vivo. At 12 weeks after supravalvular AS induction, c-hβ3tg mice and their littermate controls were examined by ^18^fluorodeoxyglucose positron emission tomography–computed tomography ([^18^F]FDG PET-CT) to monitor cardiac uptake of glucose (Fig. 3A). As expected, WT mice showed an overt cardiac metabolic reprogramming characterized by much higher glucose utilization than sham-treated animals. This metabolic reprogramming was not observed in c-hβ3tg mice (Fig. 3B).

Previous experimental studies showed an association between pressure overload and mitochondrial fragmentation [24, 25], and we previously showed that increased mitochondrial fragmentation leads to an increased cardiac glucose metabolism and heart failure [72]. We therefore examined mitochondrial morphology by transmission electron microscopy (TEM) in hearts harvested at the end of the 12-week AS protocol. AS with superimposed HF in WT mice was associated with smaller mitochondria with a disrupted crista architecture (Fig. 3C). In contrast, hearts from c-hβ3tg mice had larger mitochondria and a preserved crista morphology (Fig. 3C). The incidence of ultra-fragmented mitochondria in c-hβ3tg mice was half that of their littermate controls (Fig. 3D–F). Moreover, protein expression of master regulators of mitochondrial fusion (OPA-1 and mitofusin-2) was significantly higher in c-hβ3AR mice than in controls (Fig. 3G–H), with no changes being observed in the absence of TAC, as well as an attenuation of UCP2 (Fig. S8). These outcomes are consistent with a reduction in mitochondrial fission and an increase in ATP production.

### Gene-therapy–mediated cardiac β3AR overexpression protects against aortic-stenosis–induced left ventricular hypertrophy and heart failure

Having demonstrated the ability of constitutive β3AR overexpression to prevent the deterioration in cardiac function and metabolism provoked by AS, we next tested the translational potential of this strategy. For this approach, we drove therapeutic overexpression of human β3AR by injecting hβ3AR-encoding AAV into adult mice. The recombinant cTnT-driven hβ3AR construct was the same as that used in the rat cardiomyocyte AAV experiments (Fig. S1A, S1B), but this time packed in AAV serotype 9, which efficiently transduces mouse cardiac tissue [53] and which we previously showed is a potent tool for gene therapy in mice when used in combination with a cTnT promoter [16]. Wild-type C57Bl6J mice received 3 × 10^11^ viral particles by IV injection into the femoral vein (Fig. S9A-S9C).

To assess the efficacy of AAV9-mediated hβ3AR expression, we first monitored hβ3AR mRNA by RT-PCR in cardiomyocytes isolated from mice 4 weeks after AAV9-hβ3AR injection, using primers designed to target human β3AR but not mouse β3AR (Fig. S10A). The presence of hβ3AR protein in cardiomyocytes was verified by immunostaining, which confirmed localization in the sarcolemma (Fig. S10B), indicating correct posttranslational modification and processing necessary for membrane insertion. hβ3AR protein function was assessed by two approaches. Ex vivo experiments examined β3AR-mediated activation of sGC [6], revealing higher β3AR-agonist–stimulated cGMP production in hearts from AAV9-hβ3AR–transduced mβ3KO mice than in controls (Fig. S10C). Invasive in vivo analysis of LV function in anesthetized AAV9-hβ3AR–transduced WT mice detected a stronger β3AR-agonist–stimulated increase in dP/dt_max_ than in AAV9-EGFP–transduced controls and a trend toward higher LVESP (Fig. S10D, 6E), as well as increased dP/dt_min_ (Fig. S10F). β3AR-agonist stimulation also induced a stronger increase in heart rate in AAV9-hβ3AR–transduced mice (Fig. S10G). These data confirm that AAV9-delivered hβ3AR increases cardiac inotropy and chronotropy in mice, supporting the results obtained in c-hβ3tg mice.

**Fig. 4:**
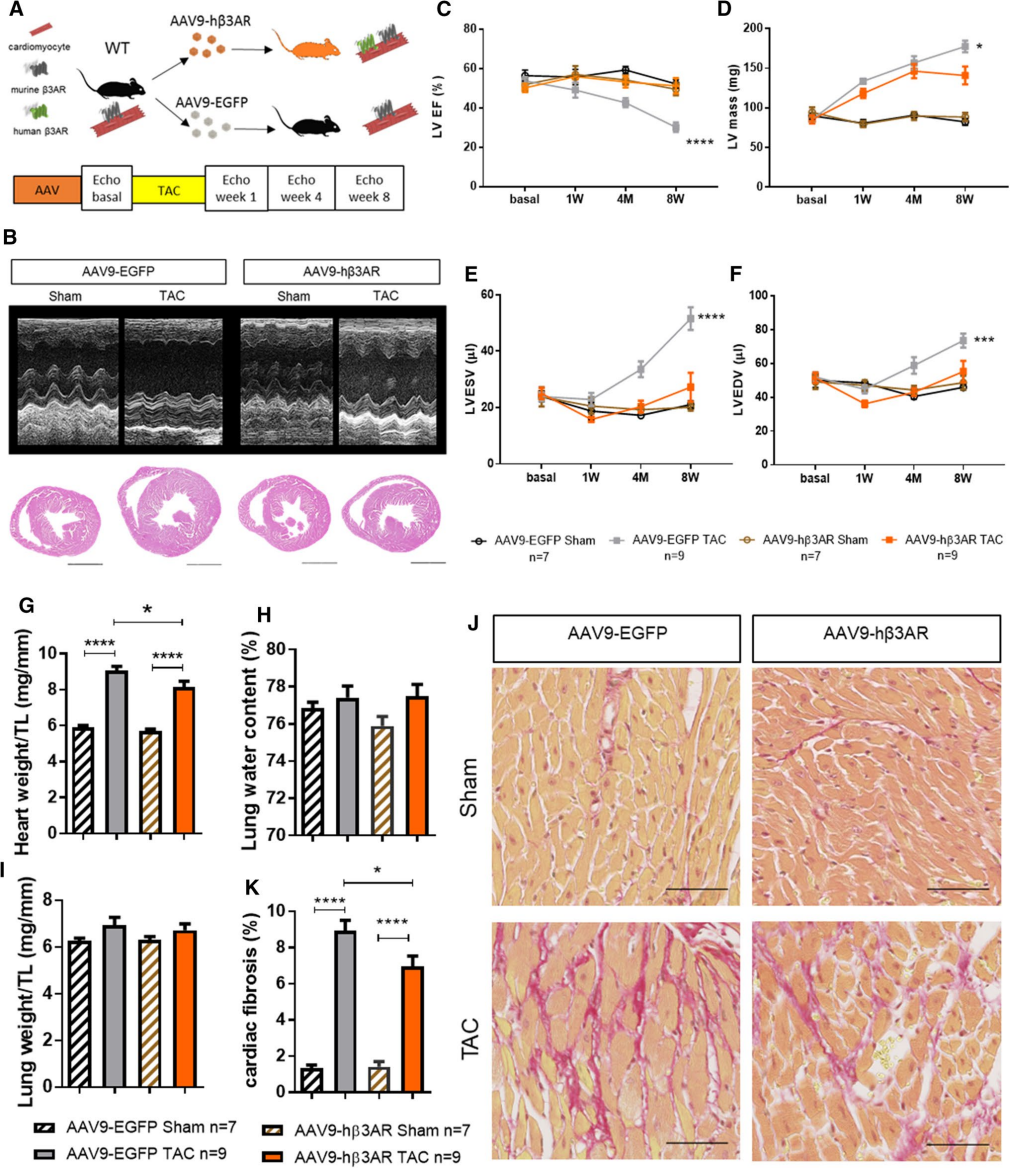
Cardiac AAV-mediated β3AR overexpression protects against aortic-stenosis–induced left ventricular remodeling and heart failure. **A** C57Bl6J WT mice were transduced with 3 × 10^11^ viral genomes per mouse of AAV9-hβ3AR (orange) or control AAV9-EGFP (gray); mice were subjected to TAC or sham surgery after 4 weeks and were followed for a further 8 weeks. **B** Representative left ventricle M-mode echocardiograms (upper panels) and heart sections (lower panels) 8 weeks after surgery. Scale bar, 2 mm. **C**–**F** Echocardiographic assessment of left ventricular ejection fraction (LVEF), left ventricular mass, and left ventricular internal volume in systole (LVESV) and diastole (LVEDV). Data are means ± SEM (*n* = 7–9). Two-way ANOVA, **p < 0.01, ***p < 0.001, ****p < 0.0001 vs TAC-operated AAV9-hβ3AR mice. G Heart weight normalized to tibia length (TL) at 12 weeks after AAV injection (8 weeks post-surgery) in TAC-operated and sham-operated AAV9-hβ3AR and AAV9-EGFP mice. **H** Lung weight normalized to TL. I Lung water content as a percentage of fresh lung weight. **J** Fibrosis and cardiomyocyte size. Histological images of heart sections show staining with the fibrosis marker sirius red (SR) and hematoxylin–eosin (HE). Scale bar, 50 μm. **K** Quantification of the total area of fibrosis in heart sections. Data in **G**–**I** and **K** are means ± SEM (*n* = 7–9). Two-way ANOVA with Tukey’s multiple comparisons test. *p < 0.05, ****p < 0.0001

We next studied the preventive effect of AAV-mediated stable hβ3AR overexpression on aortic-stenosis–induced HF. WT mice were transduced with 3 × 10^11^ viral genomes/mouse of AAV9-hβ3AR or control AAV9-EGFP, and supravalvular AS was induced 4 weeks later (Fig. 4A). Mice were followed up for 8 weeks with serial echocardiographic evaluations (Fig. 4B). Like the transgenic mice constitutively overexpressing hβ3AR, AAV9-hβ3AR mice were protected against AS–induced HF. Although LVH increased, LV volumes and LVEF were unaffected in mice with AAV-mediated hβ3AR overexpression, whereas controls developed severe LVH and HF (Fig. 4C–F). The trajectory of LVH in AAV9-hβ3AR mice featured a modest increase in LV mass that was attenuated toward the end of the follow-up period, suggesting that hβ3AR overexpression protects against maladaptive remodeling (Fig. 4D). Postmortem examination at end follow-up showed that heart weight was lower and myocardial fibrosis less pronounced in AAV9-hβ3AR mice than in AAV9-EGFP controls (Fig. 4G–K).

### AAV-β3AR gene therapy rescues the aortic-stenosis–induced failing heart

**Fig. 5:**
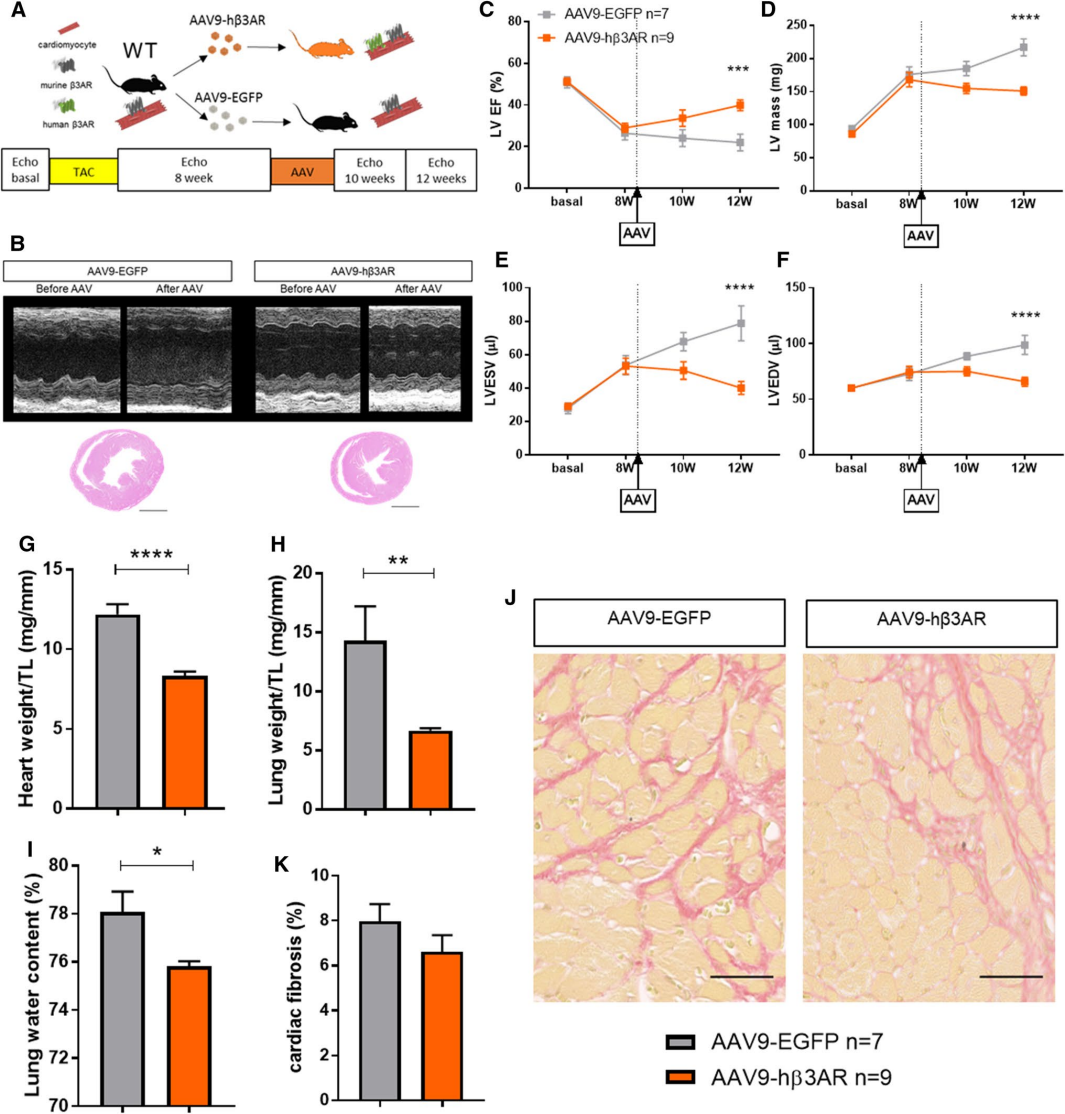
AAV-β3AR gene therapy rescues the AS–mediated failing heart by improving cardiac function and preventing further ventricular dilation in decompensated heart failure. **A** C57Bl6J WT mice underwent TAC surgery. After 8 weeks, mice were transduced with AAV9-hβ3AR (orange) or control AAV9-EGFP (gray) and were followed for a further 4 weeks. **B** Representative left ventricle M-mode echocardiograms (upper panels) and heart sections (lower panels) 12 weeks after surgery. Scale bar, 2 mm. **C**–**F** Echocardiographic evaluation of left ventricular ejection fraction (LVEF), left ventricular mass, and left ventricular internal volume in systole (LVESV) and diastole (LVEDV). Data are means ± SEM (*n* = 7–9). Two-way ANOVA with Sidak’s multiple comparisons test. ***p < 0.001, ****p < 0.0001. G Heart weight normalized to tibia length (TL) at 4 weeks after AAV injection (12 weeks post-surgery) in TAC-operated and sham-operated mice injected with AAV9-hβ3AR or AAV9-EGFP. **H** Lung weight normalized to TL. **I** Lung water content as a percentage of fresh lung weight. **J** Fibrosis and cardiomyocyte size. Histological images of heart sections show staining with the fibrosis marker sirius red (SR) and hematoxylin–eosin (HE). Scale bar, 50 μm. K Quantification of the total area of fibrosis in heart sections. Data in **G**–**I** and **K** are means ± SEM (*n* = 7–9). Student *t*-test. *p < 0.05, **p < 0.01 ****p < 0.0001

To assess the therapeutic potential of AAV-β3AR gene therapy, we randomized WT mice to AAV9-hβ3AR or control AAV9-EGFP injection 8 weeks after supravalvular AS induction, by which time, mice have developed pronounced LVH and cardiac dysfunction (LVEF < 40%), and followed them for an additional 4 weeks (Fig. 5A). Control mice (injected with AAV9-EGFP) showed a progressive increase in LVH with poor evolution of HF markers to the end of the protocol, including increased LV volume and decreased LVEF. Conversely, AAV9-hβ3AR injection protected mice against further LV dilation and significantly improved cardiac function evaluated by LVEF (Fig. 5C–F). At the end of the 12-week protocol, hearts from AAV9-hβ3AR mice were significantly lighter than AAV9-EGFP controls (Fig. 5G). β3AR gene therapy also significantly lowered lung weight and water content (Fig. 5H and I). Fibrosis analysis showed no significant changes between groups (Fig. 5J and K). Despite the overt improvement in cardiac function, AAV9-hβ3AR-injected mice showed no differences from controls in glucose uptake evaluated by end-protocol ^18^FDG PET (Fig. S11).

These data show that AAV9-hβ3AR gene therapy reverses AS–induced myocardial dysfunction. Mice injected with AAV9-hβ3AR had a significant recovery of cardiac function and showed a trend toward regression of LVH and ventricular volumes. The therapy reduced lung congestion, demonstrating rescue from overt HF.

### AAV-mediated cardiac β3AR overexpression is associated with balanced mitochondrial dynamics

**Fig. 6:**
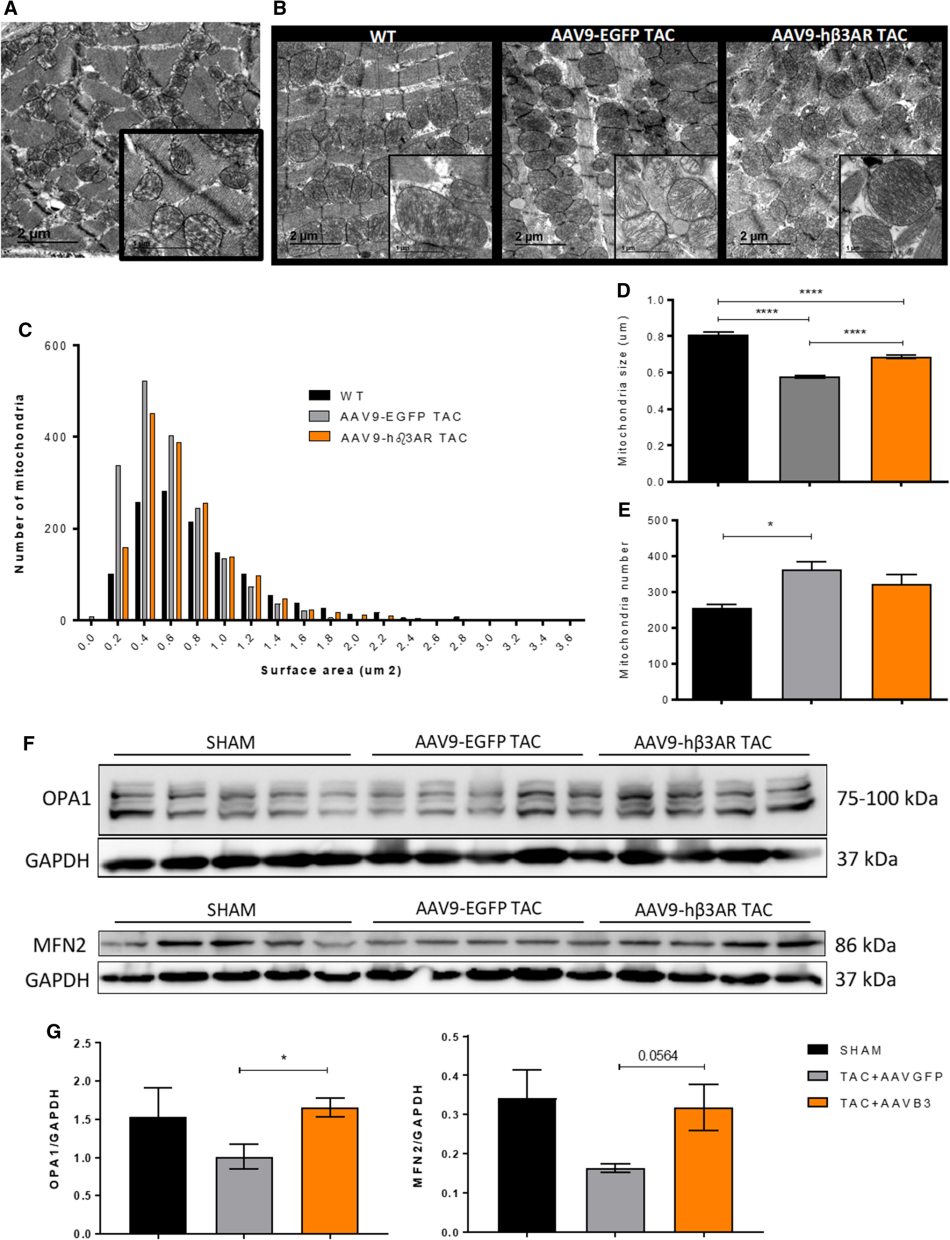
Changes in mitochondrial structure and dynamics at 2 months post-TAC surgery are improved 1 month after AAV9-hβ3AR therapy. **A** Representative cardiac transmission electron microscopy from a 2-months-post-TAC WT mouse. **B** Similar images from sham animals (WT), animals injected at 2 months post-TAC with AAV9-EGFP or AAV9-hβ3AR and examined 1 month later. **C** Frequency distribution of mitochondrial surface area in hearts of animals treated as in (**B)**. **D** Mean mitochondrial surface area. **E** Mean mitochondrial number. Data in **D** and **E** are means ± SEM of *n* = 1500–2000 mitochondria (*n* = 5 animals). One-way ANOVA follow by Tukey’s multiple comparisons test. *p < 0.05, ****p < 0.0001. **F** Western blot analysis of OPA1 and MFN2 expression in hearts of animals treated as in **B** (*n* = 5). GAPDH was used to normalize protein loading. **F** Densitometric comparison of mean OPA1 and MFN2 protein expression. Data are means ± SEM (*n* = 5). Unpaired Student *t*-test, *p < 0.05

At 8 weeks after supravalvular AS induction and before the gene therapy intervention, mouse hearts showed imbalanced mitochondrial dynamics characterized by fragmented mitochondria (Fig. 6A). AAV9-hβ3AR injection rebalanced mitochondrial dynamics, whereas animals injected with control AAV9-EGFP maintained the mitochondrial fragmentation phenotype (Fig. 6B–E). Moreover, cardiomyocyte mitochondria from AAV9-hβ3AR-injected mice were larger and had restored expression of OPA-1 and mitofusin-2 (Fig. 6F and G).

**Fig. 7:**
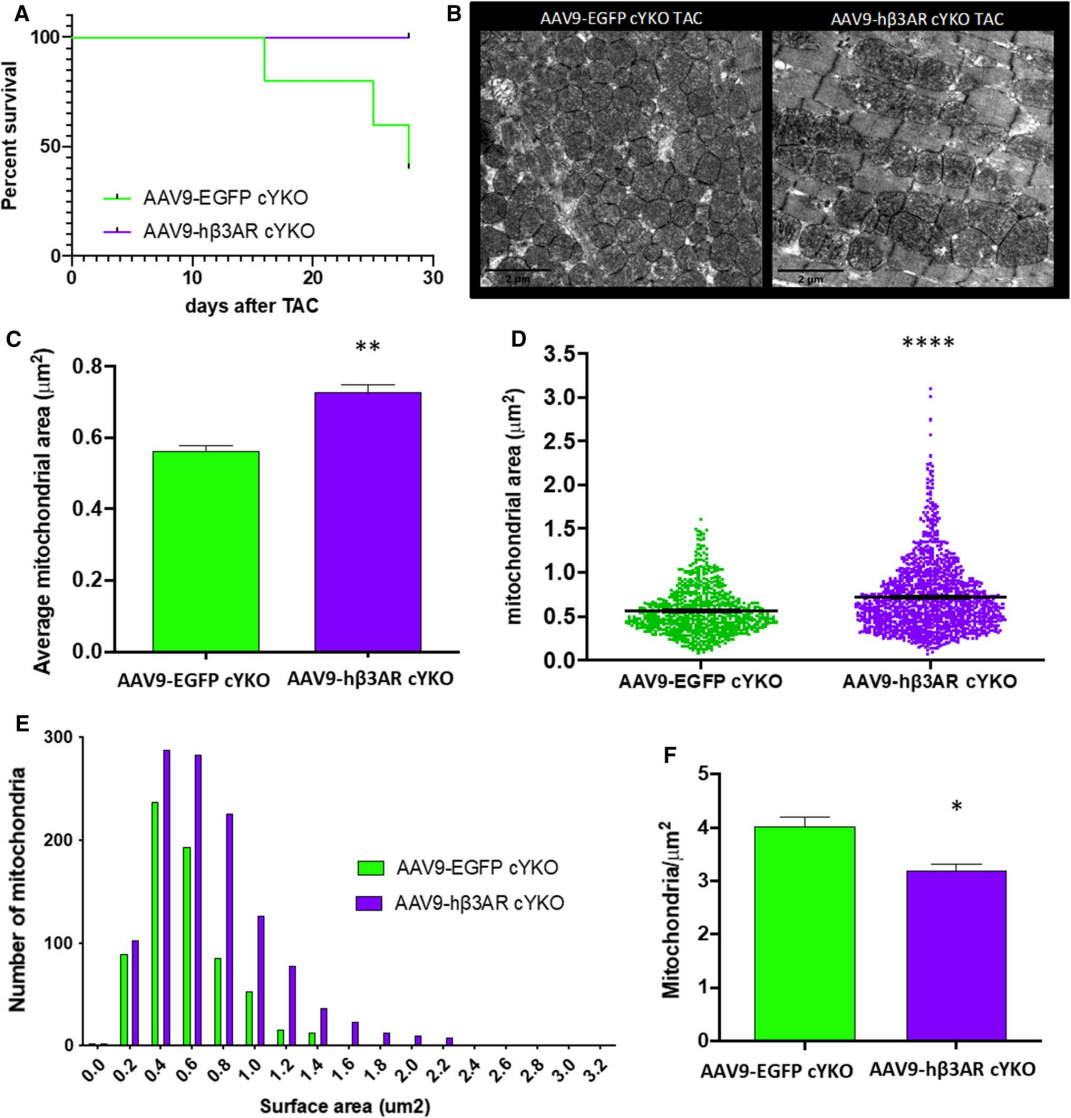
Improvement in survival and mitochondrial structure and dynamics 1 month post-TAC surgery in cYKO treated with AAV9-hβ3AR therapy. **A** Percentage of survival after TAC in cYKO mice transduced with 3 × 10^11^ viral genomes per mouse of AAV9-hβ3AR (purple, *n* = 5) or control AAV9-EGFP (green, *n* = 5) 1 month before TAC (log-rank Mantel-Cox test, *P < 0.05). **B** Representative cardiac transmission electron microscopy from cYKO mice treated as in A 1 month after TAC. **C** Mean mitochondrial surface area in hearts from cYKO mice treated as in A 1 month after TAC, (AAV9-EGFP *n* = 2 and AAV9-hβ3AR *n* = 5). Unpaired Student *t*-test, **p < 0.01. D Individual mitochondrial surface in hearts from cYKO mice treated as in A 1 month after TAC (AAV9-EGFP *n* = 689 mitochondria and AAV9-hβ3AR *n* = 1208 mitochondria). Wilcoxon-Mann–Whitney test, ****p < 0.0001. **E** Frequency distribution of mitochondrial surface area. **F** Mean mitochondrial number. Unpaired Student *t*-test, *p < 0.05. Data are means ± SEM

In order to study whether the mitochondrial dynamics restoration was involved in the mechanism leading to HF rescue by c-hβ3AR overexpression, we studied mice with pre-existing altered mitochondrial dynamics. Mice with cardiac specific *Yme1l* ablation (cYKO) display a phenotype characterized by fragmented mitochondria and dilated cardiomyopathy of late onset (20 weeks of age), as we have previously reported [72]. At 10 weeks, cardiac anatomy and function is not different from controls. At this age, cYKO mice underwent AS induction. During the first month after AS induction, a 60% mortality was observed in cYKO (Fig. 7A), while no mortality is observed in wild type mice. As expected, cYKO mice undergoing AS induction displayed altered mitochondrial dynamics with ultra-fragmented mitochondria. AAV9-hβ3AR injection before AS induction was able to rescue cYKO from death (Fig. 7A). Cardiac specific overexpression of c-hβ3AR resulted in a significant increase mean mitochondrial size on TEM (Fig. 7B–F), linking mitochondrial balance restoration to rescue from HF and death.

## Discussion

In this study, we have shown that AS–induced LVH and HF in mice are prevented by β3AR overexpression achieved by two strategies: constitutive myocardial-specific (cTnT-mediated) transgene expression of human β3AR and AAV9-mediated hβ3AR gene transfer in adult wild-type mice. Importantly, AAV9-mediated β3AR gene therapy delivered at intermediate/advanced stages of AS–induced HF was able to improve the disease. This approach has translational potential as a therapeutic strategy to halt progression or revert cardiac dysfunction in AS patients.

AS causes progressive pressure overload of the LV, triggering compensatory hypertrophy and increased contractility to maintain cardiac output. Long-lasting pressure overload can result in failure of the myocardium, which progressively deteriorates and loses contractile capacity. Untreated AS with superimposed HF or symptoms is associated with a very high annual mortality (50%) [23]. Although valve replacement resolves the pressure overload, LVH often persists and is associated with chronic HF and poor prognosis. Currently no specific treatments are able to regress LVH and prevent AS-associated HF. In the present study, we show that β3AR overexpression prevents LVH and HF secondary to induced AS and even partially reverts cardiac deterioration at advanced disease stages. The success in our study of translational AAV-mediated gene therapy demonstrates the therapeutic potential of this approach for different stages of AS. AAV gene therapy has been used in several clinical trials to treat several targets in HF [31–33, 56–58, 77] and has been shown to be safe. We propose that AAV-β3AR could be used at intermediate stages of AS to halt the progression of LV impairment and also at advanced disease stages simultaneously or after valve replacement to revert LV deterioration and prevent or treat HF.

The impact of β3AR signaling on myocardial function has been a matter of debate. Two previous studies examined the role of β3AR in distinct transgenic mouse models with constitutive cardiac hβ3AR expression driven by the myosin heavy chain (MHC) promoter. These studies produced contradictory results: Kohout et al. described a positive inotropic effect of β3AR signaling in in vivo experiments [36], whereas Tavernier et al. showed a negative inotropic effect in ex vivo cardiac tissue exposed to β3AR agonists [70]. The conflict between the results of these studies may be due to differences between the in vivo and ex vivo experimental models. In addition, an important limitation of both studies is that hemodynamic effects of hβ3AR stimulation were studied in animals expressing human and mouse β3AR. This is important because the affinity of β3AR agonists differs between the mouse and human receptors. To overcome this limitation, we crossed c-hβ3AR mice (with β3AR driven by the cTnT promoter) with β3AR KO mice, yielding mice expressing only human β3AR in the myocardium. This strategy allowed us to study the role of human β3AR in cardiomyocytes without interference from mouse β3AR background expression in the heart or other cell types contributing to hemodynamic reactions (smooth muscle cells, endothelium, etc.).

In our transgenic model, stimulation of cardiomyocyte-expressed hβ3AR with the human β3AR-selective agonist mirabegron produced a positive chronotropic and inotropic effect in vivo, in line with previously reported by other authors [42]. The same positive inotropic response to the β3AR stimulation was also observed in wild-type mice overexpressing hβ3AR by AAV gene therapy. In another study of cardiac tissue, the inotropic effect of hβ3AR stimulation was not detected; however, that analysis was performed ex vivo [46]. Intriguingly, in vivo β3AR stimulation in sheep has a negative inotropic effect in physiological conditions and a positive inotropic effect in HF [11], suggesting that increased numbers of the receptor may affect its coupling. In our study, hβ3AR-mediated inotropy is supported not only by the physiological analysis, but also by the increase in LVEF in transgenic mice at week 1 after AS induction.

Intense debate has surrounded the question of whether β3AR upregulation in clinical HF contributes to the disease or is instead a protective mechanism, and a variety of animal models has been used to investigate this. β3AR signaling was suggested to contribute to HF progression in the dog [47], and chronic β3AR blockade was reported to improve cardiac function in a rat model [20]. However, many other studies have shown a beneficial effect of β3AR signaling in HF, positioning it as a potential therapeutic target [6, 45, 49, 73]. Our study confirms the beneficial effect, showing that specific β3AR overexpression in cardiomyocytes boosts NO/cGMP over cAMP/PKA signaling [15, 28, 44, 66, 74] and prevents HF and mitochondrial dysfunction in a mouse model of AS.

Recent studies have demonstrated that specific distributions and expression of βAR in microdomains are essential for the coupling to signaling effectors, as well as for the regulation of intracellular mediator pools and their diffusion patterns [17, 55]. This tight-regulated phenomenon attributes distinct effects to a given mediator (e.g. cAMP) and could explain the divergent actions of βAR subtype signaling in healthy/pathological conditions and thus physiological effects. Besides, communication among intracellular mediators is also crucial for an appropriate regulation. In this context, cGMP boost and modulation of cAMP levels through a PDE2-based cGMP-to-cAMP crosstalk [67] could also explain the increase in cGMP:cAMP ratio in β3AR overexpressed cardiomyocytes.

In our model, serial echocardiography revealed better systolic function (preserved LVEF) in c-hβ3tg mice throughout the experiment, a result confirmed by invasive LV pressure evaluation at the end of the experiment. During the first week after surgery, c-hβ3tg mice showed features of compensatory concentric hypertrophy, such as lower LV volumes and elevated LVEF, suggesting that endogenous catecholamines activate the hβ3AR to generate a positive inotropic response similar to that produced in non-operated animals by pharmacological challenge with a specific β3AR agonist. c-hβ3tg mice were consistently protected throughout the 12-week experimental protocol; however, animals pre-treated with L-NAME exhibited a poor survival after AS, thus pointing towards a β3AR-associated protection through NO/cGMP signaling. More importantly, the anti-HF effects of this strategy are reinforced by the consistently lower lung mass and pulmonary edema in c-hβ3AR mice. The long-term cardioprotective effect of β3AR overexpression is likely due to the lack of phosphorylation sites in the receptor, making it less prone to desensitization and downregulation [43] than other βAR subtypes [26, 59]. Echocardiography and histology also demonstrated attenuation of adverse cardiac remodeling in c-hβ3tg mice. Our results are in agreement with a previous study of mice lacking β3AR, which showed an age-dependent increase in cardiac hypertrophy and a more severe LV remodeling in response to pressure overload [45]. Moreover, another study showed that treatment with a β3AR agonist attenuated LV dilation and systolic dysfunction and partially reduced pressure-overload–induced LVH [49]. The beneficial effects of hβ3AR overexpression on LV fibrosis and cardiomyocyte hypertrophy seen in our study are in line with studies that explored the antihypertrophic and antifibrotic effect of β3AR in another mouse model with cardiomyocyte-specific overexpression [6, 27].

Our study is the first to show a therapeutic effect of β3AR overexpression at advanced stages of HF. AAV9-hβ3AR injection before AS induction confirmed the similar beneficial effects of AAV-mediated and constitutive transgenic cardiac β3AR overexpression. Therapeutic AAV9-hβ3AR injection at advanced stages of induced AS (featuring LVH and incipient signs of HF) prevented further deterioration of cardiac function and even partially reverted the negative LV remodeling present at the time of treatment. The AAV9-mediated gene therapy approach has two main advantages: it results in stable gene overexpression in nondividing cells like cardiomyocytes, and it has high translational potential since AAV it has been shown to effective and safe in several clinical trials, including in HF patients [31–33, 77]. Gene therapy has been widely used in mouse models to overexpress proteins with demonstrated beneficial effects [57, 63]. Recombinant AAVs are among the most used vectors for this purpose due their safety, long-term transgene expression, and the flexibility afforded by varied tropisms of the different serotypes. The ability of AAV-mediated hβ3AR gene therapy to revert disease even when injected late in its course is extremely important because clinical therapy will only be initiated upon the appearance of disease symptoms, such as LVH or HF in the present context.

Our results shown that β3AR overexpression protection is associated with an effect on cardiac metabolism and mitochondrial function. The ^18^FDG PET/CT studies revealed metabolic reprogramming from fatty acids to glucose in hearts from WT mice with aortic-stenosis–induced HF, and the fragmented cardiomyocyte mitochondria in these animals indicated misbalanced mitochondrial dynamics. Mitochondrial fragmentation and metabolic reprogramming were prevented by β3AR overexpression present at the time of AS induction, despite the persistence of a degree of LVH. These results were also consistent with an increase in ATP in β3AR-overexpressed hearts at early stages post-AS induction. In adipocytes, β3AR signaling has been linked to increased mitochondrial density as a mechanism to sustain the higher metabolic demands associated with the switch from white to the beige phenotype [5, 65]. Pressure overload generated after AS induction is associated with higher metabolic demands on cardiomyocytes [38], and it is thus plausible that β3AR overexpression enables these cells to meet these higher demands by preserving mitochondrial function and thus preventing the onset of HF. Our results are in line with a vast literature showing the association of HF with a metabolic switch from fatty acids to glucose as the principal energy source [35, 52], and metabolic reprogramming has been previously documented in mice with induced AS [37]. We previously showed that excessive mitochondrial fragmentation in dilated cardiomyopathy-induced HF impairs mitochondrial function, contributing to the metabolic reprogramming [72]. In the present study, c-hβ3tg mice with induced AS had fewer ultra-fragmented mitochondria than controls, and their mitochondria had more organized cristae, suggesting that they were healthier and therefore better able to cope with the pressure overload. Increased cardiomyocyte fatty-acid use is reported to protect cardiac metabolism and function. For example, cardiac-specific deletion of acetyl CoA carboxylase 2, an enzyme that blocks fatty-acid entry into mitochondria, prevents the cardiac metabolic switch in a pressure-overload model and attenuates LVH and fibrosis [37]. Similarly, a recent study showed that the heart is protected against pressure-overload–induced HF by increased fatty-acid uptake induced by dietary means or by increased expression of the fatty-acid translocase CD36 in cardiomyocytes [25]. Consistent with these findings, our respirometry experiments showed that β3AR activation increases fatty-acid oxidation in a dose-dependent manner in isolated adult cardiomyocytes.

In the HF gene therapy experiments, AAV9-hβ3AR was administered after the appearance of mitochondrial fragmentation. The recovery of mitochondrial size and upregulated expression of master regulators of mitochondrial fusion (OPA1 and mitofusin-2) evidenced the restoration of normal mitochondrial dynamics. However, myocardial glucose utilization remained high in AAV9-hβ3AR-treated mice at the end of the study, despite the clear improvement in cardiac function. This dissociation between mitochondrial dynamics and substrate use can be interpreted in two ways. The most logical explanation is that substrate use is a marker of cardiac metabolism and is not necessarily involved in the recovery of cardiac function. Alternatively, β3AR overexpression may increase β-oxidation of fatty acids without affecting glucose use, which is already high at the time of gene therapy. This would lead to a general increase in cardiomyocyte metabolism and normalization of the fatty-acids–to–glucose ratio. Given that we were unable to directly measure fatty-acid use by PET, this possibility remains a matter of speculation at present. Nevertheless, all our results point to a preservation/restoration of balanced mitochondrial dynamics as the leading mechanism driving the benefits of β3AR overexpression in aortic-stenosis–induced HF. cYKO mice, which are characterized by mitochondrial fragmentation, show a high mortality rate upon AS challenge, and c-hβ3AR overexpression by AAV9 injection was able to rescue them from AS-induced death. The fact that AAV9-hβ3AR injection resulted in a significant increase in mean mitochondrial size in cYKO mice strongly suggest that restoration of balanced mitochondrial dynamics is involved in the benefits associated with cardiac β3AR overexpression in the context of AS-induced HF.

Herein we provide robust data highlighting the relevance of c-hβ3AR overexpression as a potential strategy to protect the heart from the development of HF and against cardiomyocyte mitochondrial dysfunction. Therefore, our results support other studies showing the beneficial effects of β3AR overexpression [6, 18, 27], and emphasize the need for current interventional trials to probe clinical benefits and mechanistically explore the advantageous effects of β3AR stimulation against adverse myocardial remodeling in HF [3, 60], so as to overcome limitations of previous ones [9]. Furthermore, our study proposes strong preclinical outcomes of AAV-mediated hβ3AR gene therapy as a translational strategy to protect and/or rescue the heart at different stages of disease. The translational potential of enhanced β3AR signaling is highlighted by the results of the recently published BEAT-HF trial. This pilot trial enrolled patients with chronic HF and moderate to low LVEF, who were randomized to the β3AR agonist mirabegron or placebo [9]. While the main outcome of the trial (6 months change in LVEF between groups) was neutral, mirabegron treatment was associated with a significant increase in LVEF (as compared to placebo) in the subgroup of patients with more advanced HF [9]. In line with this, a recent trial showed that 1-week oral mirabegron treatment increased cardiac index and decreased pulmonary vascular resistance in this subset of patients [10]. We speculate that the benefits observed in this subgroup are secondary to a marked overexpression of β3AR in subjects with severe HF (as opposed to those with mild disease). The latter is supported by the threefold increase in β3AR expression in explanted hearts from humans with very severe LVEF deterioration [46].

## Supplementary Information

Below is the link to the electronic supplementary material.

Supplementary file1 (DOCX 3672 KB)

## Data Availability

Data are available from the corresponding author upon reasonable request.
