# Supplementary material for: Beta-3 adrenergic receptor overexpression reverses aortic stenosis–induced heart failure and restores balanced mitochondrial dynamics
Source: Basic Res Cardiol. 2022 Nov 29;117(1):62. doi: 10.1007/s00395-022-00966-z (PMC9708808; doi:10.1007/s00395-022-00966-z)
Supplement: Supplementary file 1 — Supplementary file1 (DOCX 3672 KB) [file 395_2022_966_MOESM1_ESM.docx]

**Figure S1. Plasmids used to generate recombinant adeno-associated virus (rAAV).**


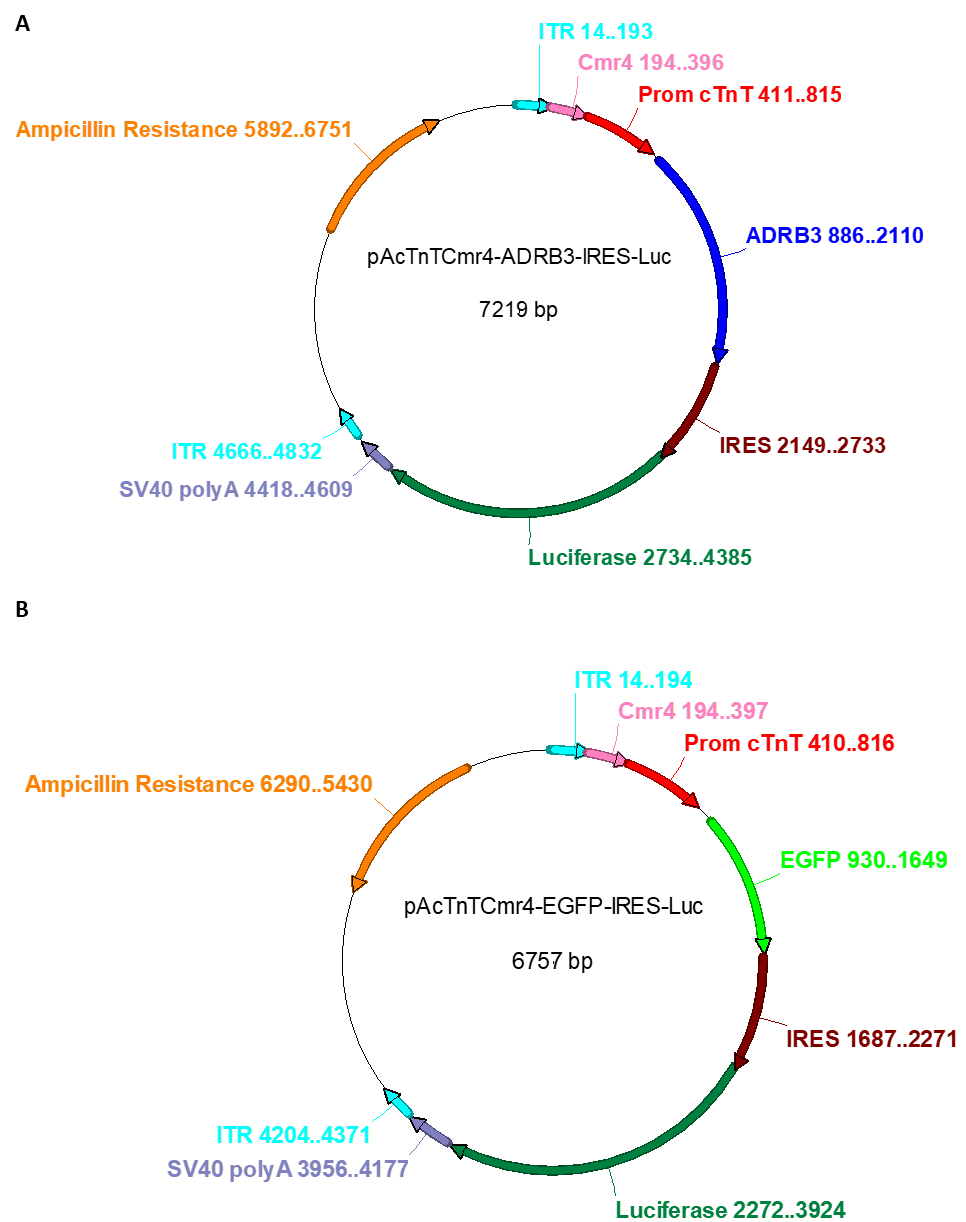


(**A**) Plasmid for hβ3AR-rAAV production. (**B**) Plasmid used for control EGFP-rAAV production. ITR, recognition site for AVV packaging; Cmr4, enhancer sequence; Prom cTnT, troponin T promoter sequence for cardiomyocyte-specific expression; ADRB3, c-DNA sequence of the human β3AR receptor; EGFP, enhanced green fluorescent protein sequence; IRES, internal ribosome entry site.

**Figure S2. Beta3-adrenergic receptor overexpression protects neonatal rat ventricular myocytes from isoproterenol-induced hypertrophy.**

**
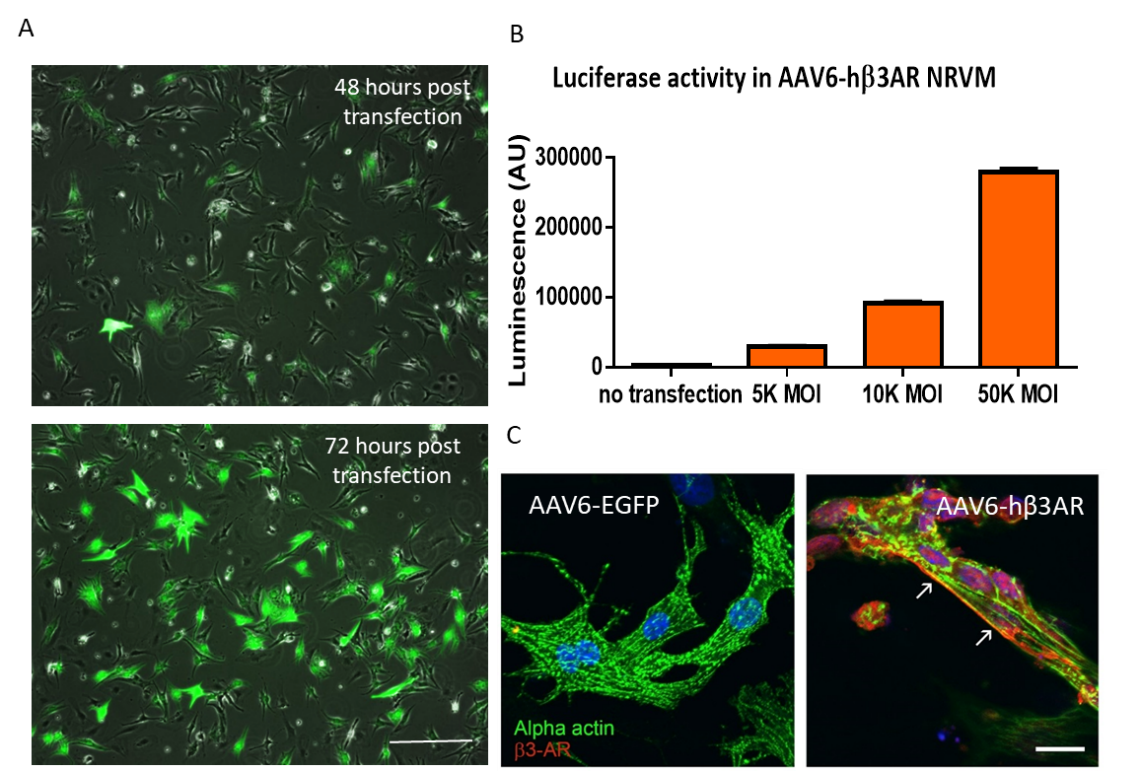
**

(**A**) Representative images of neonatal rat ventricular myocytes (NRVM) transfected with EGFP adeno-associated virus serotype 6 at 10K MOI for 48 hours (top) and 72 hours (bottom). EGFP signal is evident in most cells after 72 hours of transduction. Scale bar, 100µm. (**B**) Luciferase activity quantification in NRVMs transduced for 72 hours with hβ3AR-luciferase adeno-associated virus serotype 6 at 5K, 10K, and 50K MOI. A 10K MOI sufficiently transduces NRVMs. Data are means ± SEM from two independent experiments. (**C**) Immunostaining for α-actin (green) and hβ3AR (red) in NRVMs transduced for 72 hours with control (AAV6-EGFP) or human β3AR adeno-associated virus (AAV6-hβ3AR), showing membrane localization of hβ3AR and no signal in control cells. Scale bar, 20 µm. Data are means ± SEM.

**Figure S3. *In vivo* assessment of LV function in c-hβ3tg mβ3KO mice and mβ3KO control littermates**

**
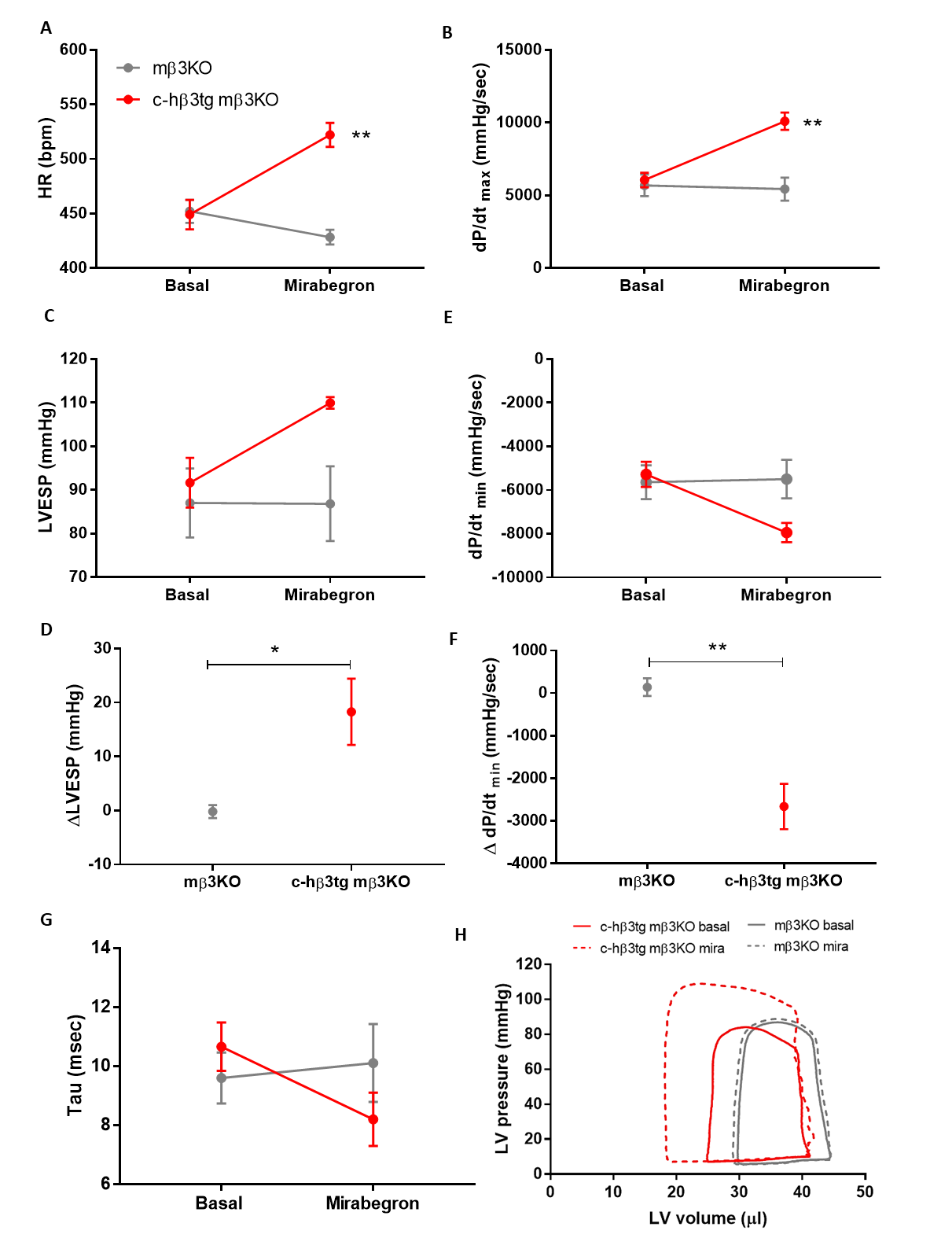
**

(**A**) Increased chronotropic response to mirabegron in c-hβ3tg mβ3KO mice indicated by the effect on heart rate (HR). (**B-D**) Increased in systolic function in c-hβ3tg mβ3KO mice: (**B**) maximal derivative of LV pressure (dP/dt_max_); (**C, D**) left ventricular end-systolic pressure (LVESP). (**E, G**) Increased diastolic function in c-hβ3tg mβ3KO mice: (**E, F**) minimal derivative of LV pressure (dP/dt_min_); (**G**) time constant of isovolumic relaxation (Tau). (**H**) Representative pressure-volume loops showing an upward and leftward shift after mirabegron administration in c-hβ3tg vs. mβ3KO mice, indicating enhanced contractility. c-hβ3tg mβ3KO mice (red, n=3) and mβ3KO control littermates (gray, n=3) at baseline and in response to the β3AR agonist mirabegron (1µg/kg). Data are means ± SEM. Two-way ANOVA, **p<0.01 and Student *t*-test (for increase plots), *p<0.05, **p<0.01.

**Figure S4. Assessment of βAR transcripts in adult cardiomyocytes.**

**
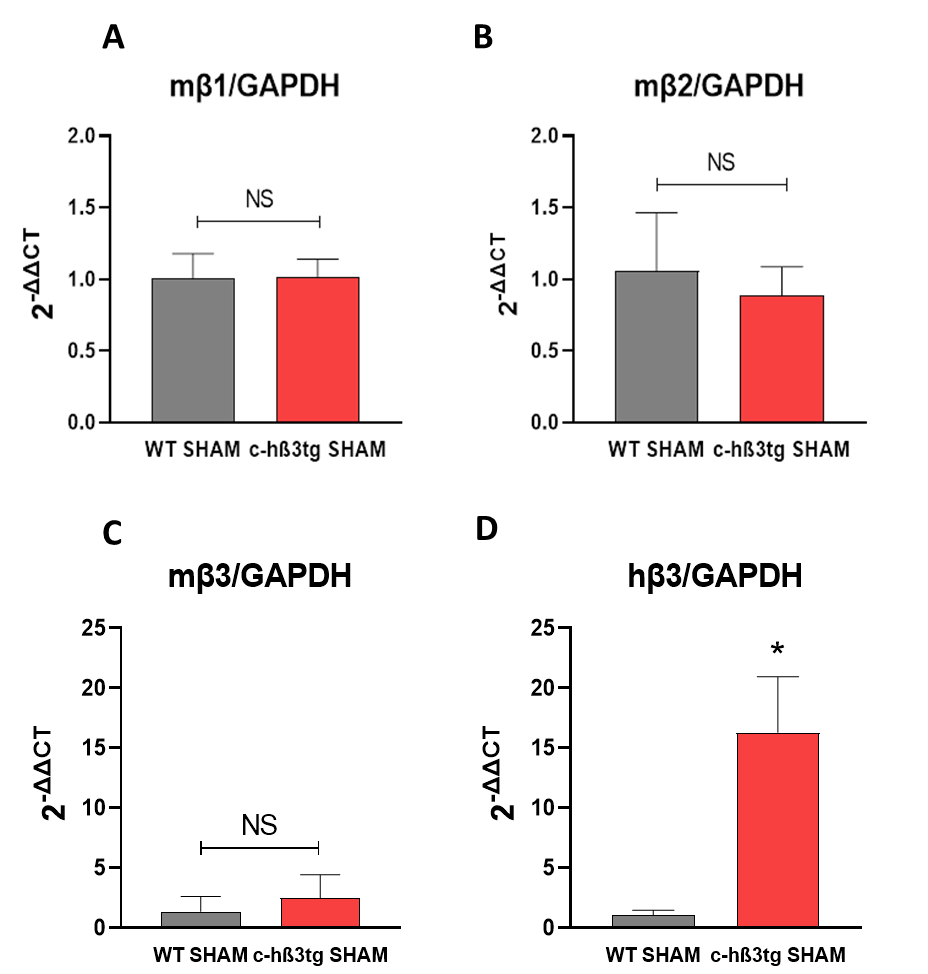
**

Evaluation of endogenous mouse *Adrb1* (A), *Adrb2* (B) and *Adrb3* (C) transcripts in the context of transgenic human β3AR overexpression (c-hβ3tg mice) by qPCR. (D) Levels of the human *Adrb3* transcript were also assessed. Data are means ± SEM. Student *t*-test or Mann-Whitney test were used to compare differences between WT (n=4) and c-hβ3tg mice (n=3). NS, not significant; *p<0.05.

**Figure S5. Adult mouse hearts perfusion and cNMPs evaluation.**

**
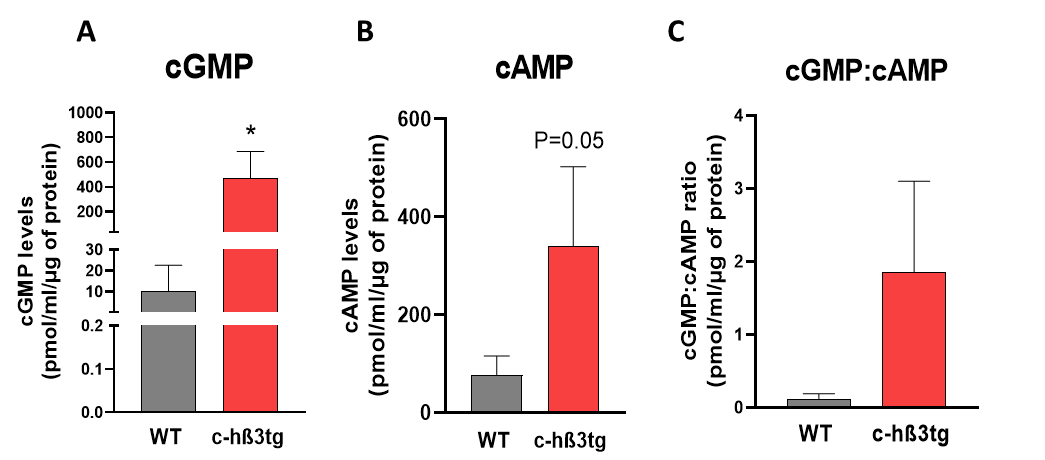
**

Hearts from c-hβ3tg and WT mice were explanted and *ex vivo* perfused with the β3AR agonist mirabegron. Upon exposure to β3AR agonist, the predominant effect was a massive increase in cGMP levels (A); however, there was a concurrent modest increase in cAMP levels (B). Therefore, cGMP:cAMP ratio was increased. Data are means ± SEM. Student *t*-test or Mann-Whitney test were used to compare differences between WT (n=4) and c-hβ3tg mice (n=3). *p<0.05.

**Figure S6. Pre-treatment with L-NAME in c-hβ3tg mice before TAC induction.**

**
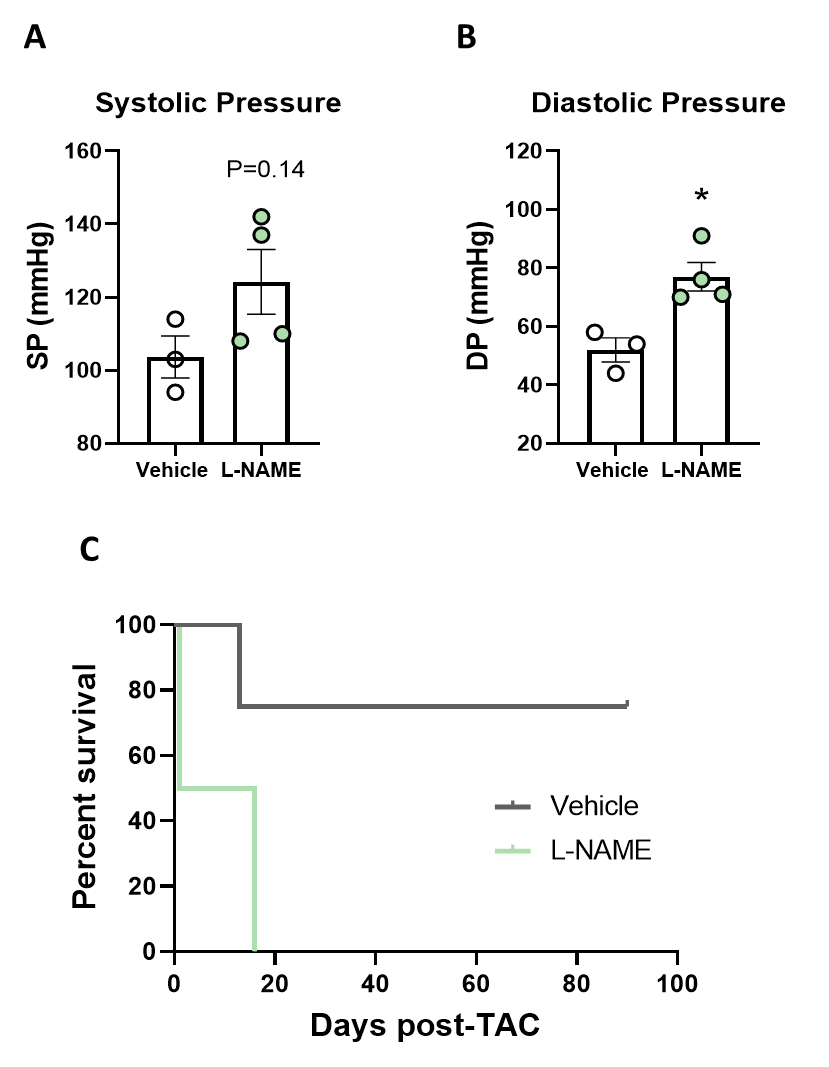
**

c-hβ3tg mice previously pre-treated with the nitric oxide synthase inhibitor L-NAME (1mg/mL) for 15d or vehicle were subjected to TAC and followed up. 15d treatment with L-NAME in c-hβ3AR mice resulted in an increase of systolic (A) and diastolic pressures (B). Data are means ± SEM. Student *t*-test was used to compare differences between vehicle (n=3) and L-NAME-treated mice (n=4). Additionally, c-hβ3tg mice under L-NAME had significantly less survival treatment than c-hβ3AR not receiving L-NAME (C). Data are shown as a Kaplan-Meier Curve. *p<0.05.

**Figure S7. Cardiomyocyte free fatty-acid and glucose utilization.**

**
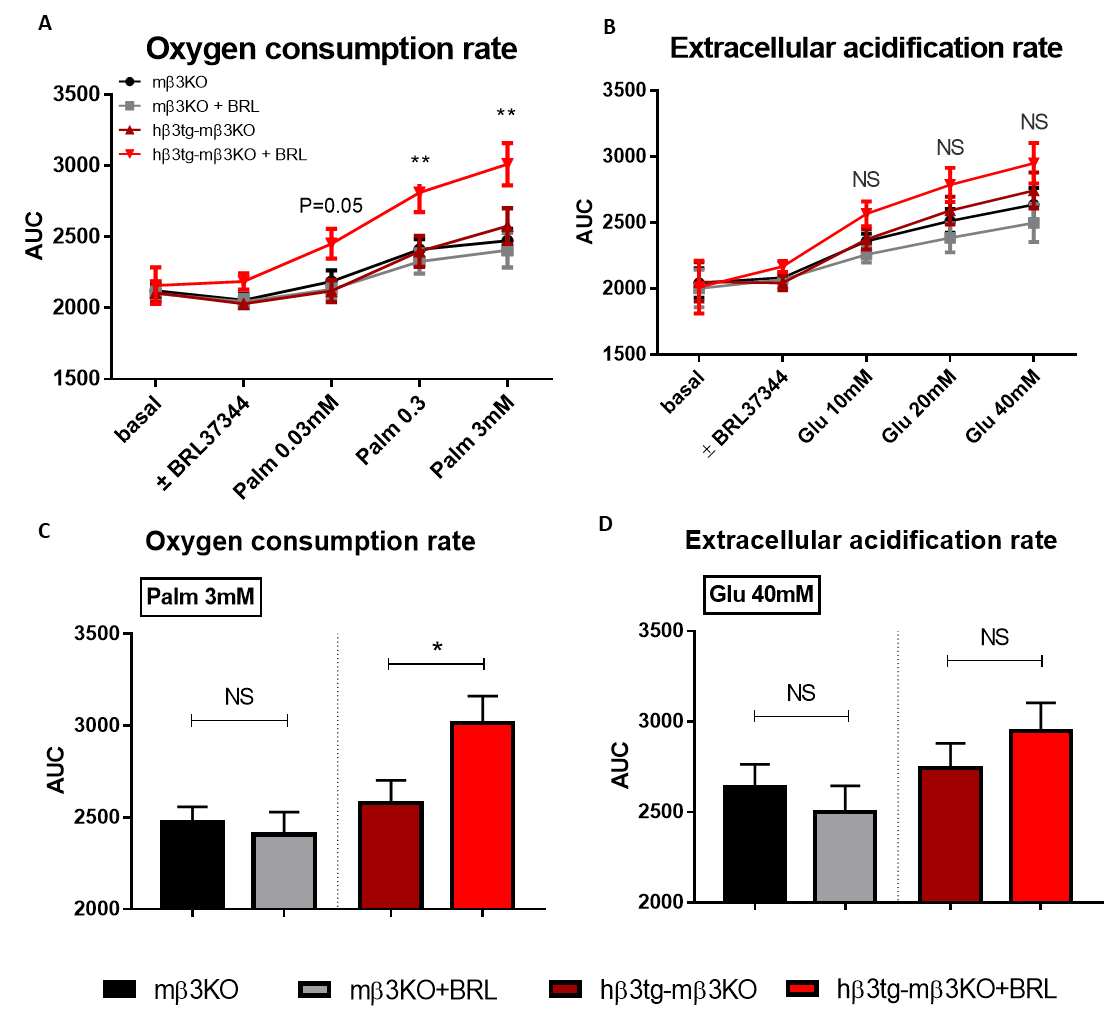
**

(A, B) Cardiomyocyte free fatty-acid utilization. (**A**) Oxygen consumption rates in cardiomyocytes isolated from healthy adult mβ3KO mice and c-hβ3tg mβ3KO mice. Cardiomyocytes were exposed to increasing doses of the free fatty acid palmitate (Palm) in the presence or absence of the β3AR agonist BRL37344 (1μM, n=8). Two-way ANOVA, **p<0.01 for c-hβ3tg mβ3KO in the presence or absence of BRL37344. (**B**) Oxygen consumption rates at the highest palmitate concentration (3mM). The elevated BRL-activated oxygen consumption rate in c-hβ3tg mβ3KO cardiomyocytes indicates increased fatty-acid utilization Student’s t test, *p<0.05. (**C, D**) Cardiomyocyte glucose utilization. (**C**) Extracellular acidification rate (an index of lactic acid production) in cardiomyocytes isolated from healthy adult mβ3KO mice and c-hβ3tg mβ3KO mice; cardiomyocytes were exposed to increasing doses of glucose (Glu) in the presence or absence of 1μM BRL37344 (n=8). Two-way ANOVA, NS, not significant, for c-hβ3tg mβ3KO in the presence or absence of BRL37344. (**D**) Extracellular acidification rates at the highest glucose concentration (40mM). Cellular metabolic parameters were measured using a Seahorse Bioscience XF96 Extracellular Flux Analyzer. Data are means ± SEM.

**Figure S8. OPA1 and MFN2 evaluation in sham-operated mice.**

**
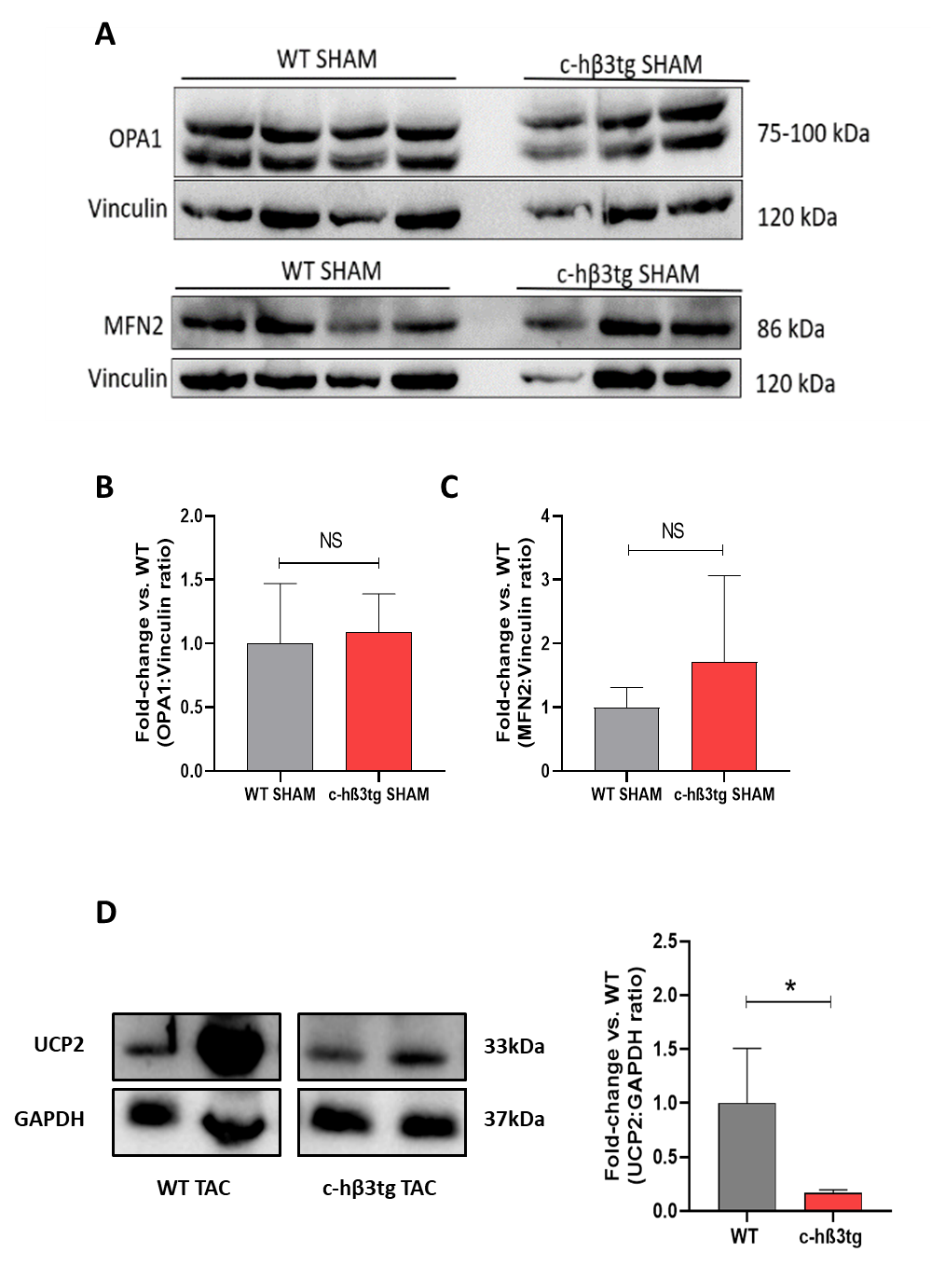
**

Protein expression of OPA1 (A, B) and MFN2 (A, C) in mice overexpressing hβ3AR (n=3) or WT (n=4) without TAC (sham) were evaluated, with no differences observed between genotypes. Expression of UCP-2 was also assessed in c-hβ3tg (n=5) or WT (n=4) 12 weeks after TAC induction. Data are means ± SEM. Student *t*-test was used to compare differences between genotypes. NS, not significant; *p<0.05.

**Figure S9. Adeno-associated virus serotype 9 (AAV9) efficiently transduces cardiac tissue in mice.**

**
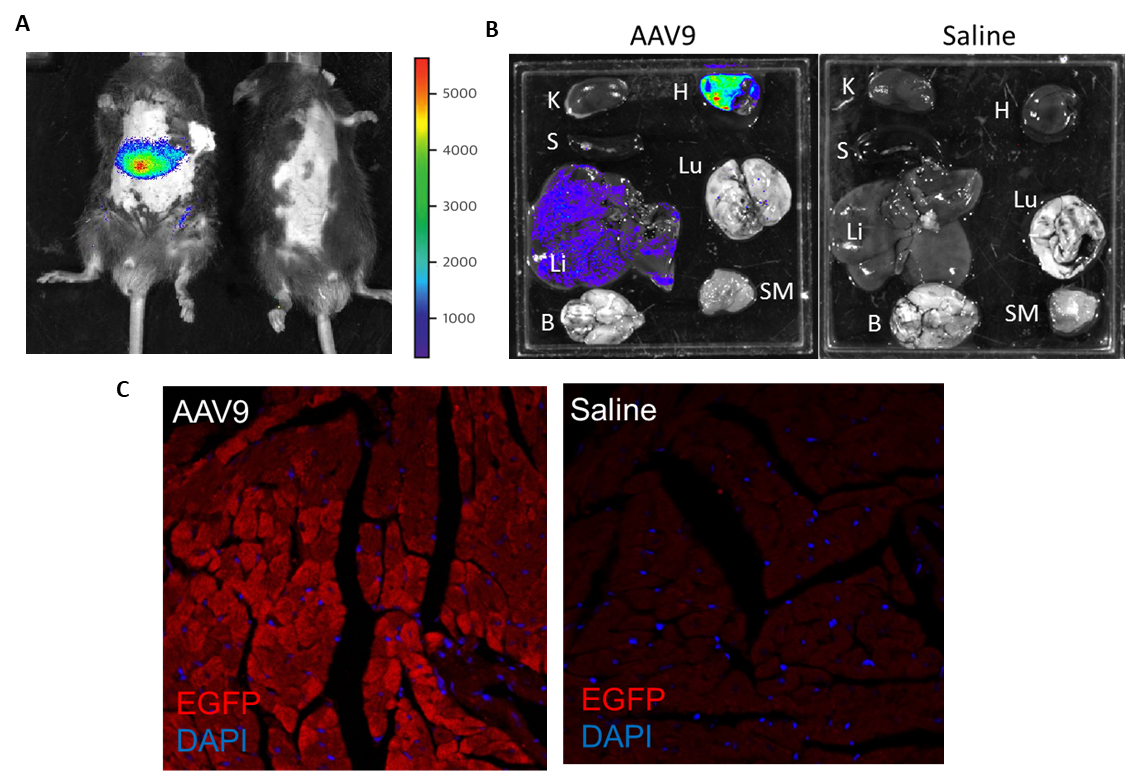
**

(**A**) *In vivo* bioluminescence images showing luciferase activity in C57Bl6J mice 2 weeks after intravenous (femoral vein) injection of AAV9 (3x10^11^ viral genomes/mouse) (left) or saline (right). (**B**) *Ex vivo* bioluminescence images from D-luciferin–organs from mice transduced with AAV9 (left) or saline (right). K, kidney; S, spleen; Li, liver; B, brain; H, heart; Lu, lungs; SM, skeletal muscle. Luciferase activity was predominantly observed the heart and activity was low in the liver. (**C**) Fluorescence microscopy of heart cryosections from mice transduced with AAV9-EGFP (left) or saline (right), showing the mosaic cell distribution of transgene expression.

**Figure S10. Mice transduced with AAV9-hβ3AR express functional beta3-adrenergic receptors in cardiomyocytes.**

**
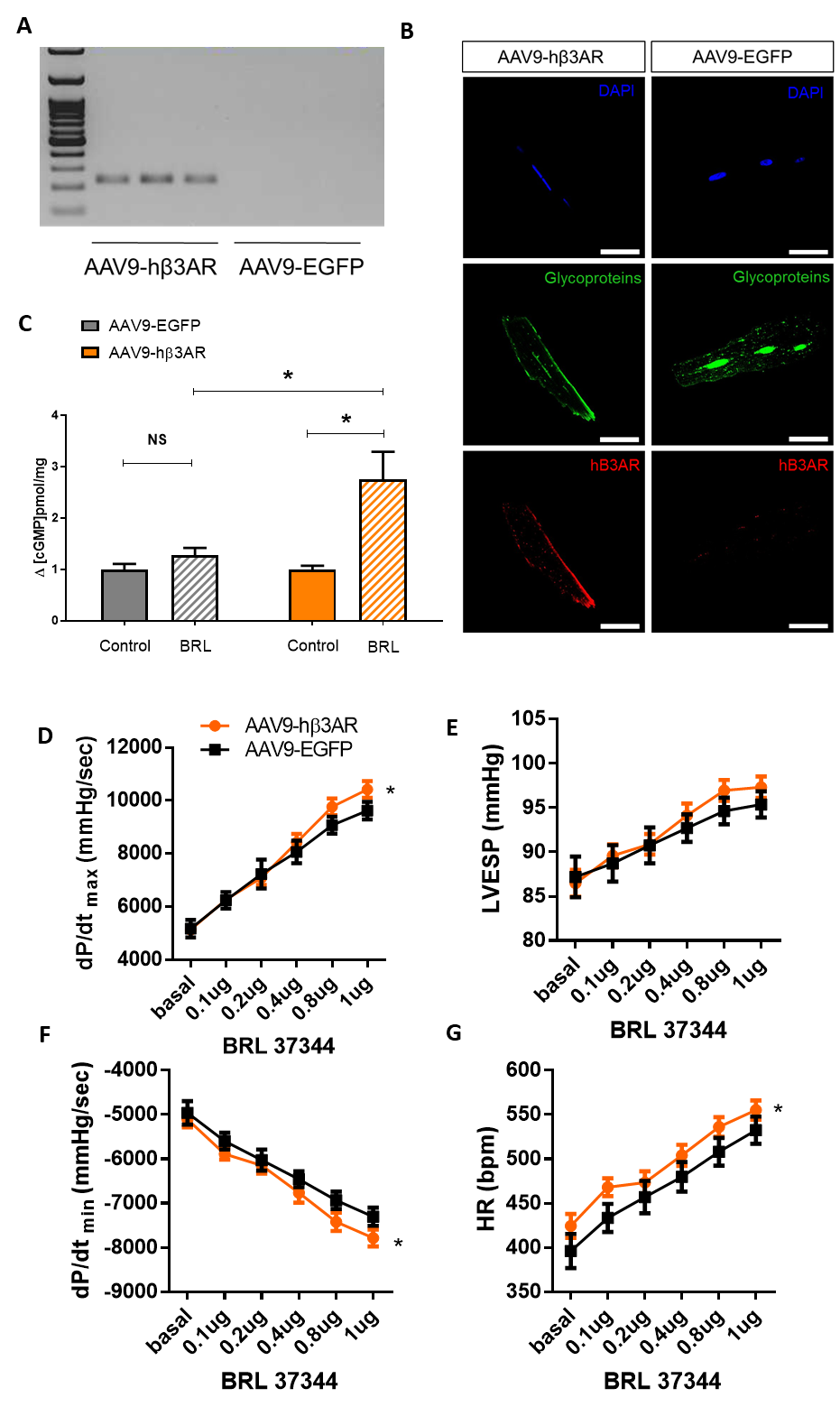
**

(**A**) RT-PCR analysis showing *ADRB3* gene expression in cardiomyocytes from mice transduced with AAV9-hβ3AR but not AAV9-EGFP. The probe was designed to target only the human β3AR cDNA sequence. (**B**) Representative immunostaining in adult mouse ventricular myocytes from mice injected with AAV9-hβ3AR (left) or control AAV9-EGFP (right). hβ3AR (red) is found at the sarcolemma of AAV9-hβ3AR-transduced cardiomyocytes. Lectin staining of membrane glycoproteins (green) is shown for comparison. Scale bar, 50µm. (**C**) β3AR-driven cGMP production in hearts from mβ3KO mice transduced with AAV9-hβ3AR or AAV9-EGFP. Hearts were perfused *ex vivo* with the β3AR agonist BRL37344 (BRL, 0.1μM) or vehicle (Control). (**D**-**G**) Analysis of left ventricular function in mice transduced with AAV9-hβ3AR (orange, n=9) or AAV9-EGFP (black, n=10) at baseline and in response to increasing doses of BRL 37344, with AAV9-hβ3AR-transduced mice showing an increased chronotropic response (heart rate, HR), increased systolic function indicated by maximal derivative of LV pressure (dP/dt_max_) and left ventricular end-systolic pressure (LVESP), and increased diastolic function indicated by the minimal derivative of LV pressure (dP/dt_min_). Data are means ± SEM. Two-way ANOVA, *p<0.05

**Figure S11. Gene-therapy– mediated beta3-adrenergic receptor overexpression in cardiomyocytes does not revert the cardiac metabolic switch in heart failure.**

**
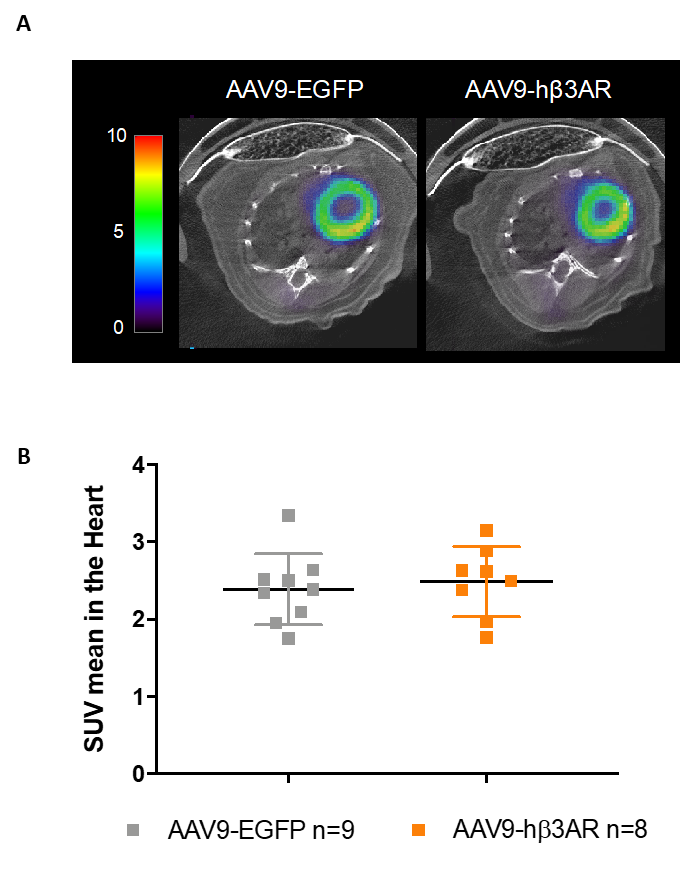
**

C57Bl6J WT mice were subjected to transaortic constriction surgery (TAC) to induce supravalvular AS. Eight weeks later, mice were transduced with AAV9-hβ3AR or control AAV9-EGFP. PET-CT scans were performed 12 weeks after surgery. (**A**) Representative PET-CT thoracic scan images after [^18^F]FDG injection. (**B**) Mean standardized uptake value (SUV). Data are means ± SEM. No significant differences were found by Student *t*-test.
